# Non-invasive biomarkers for brain aging: the role of autophagy-related microRNAs in plasma exosomes

**DOI:** 10.3389/fnmol.2025.1588007

**Published:** 2025-06-06

**Authors:** Qian Cheng, Shuyi Yu, Zhikang Cui, Hang Chen, Jing Fan, Qian Yu, Yan Jin, Yunshan Wang, Ming Li, Zhiming Lu

**Affiliations:** ^1^Department of Clinical Laboratory, Shandong Provincial Hospital Affiliated to Shandong First Medical University, Jinan, China; ^2^Shandong Provincial Hospital, Shandong University, Jinan, China

**Keywords:** brain aging, autophagy, miRNA, exosomes, biomarkers

## Abstract

**Aim:**

This study aimed to identify autophagy-related microRNAs (miRNAs) in plasma exosomes as non-invasive biomarkers for brain aging and explore their potential to improve early detection of age-associated neurodegeneration. With the increasing incidence of neurodegenerative disorders, such as Alzheimer’s disease (AD), frontotemporal dementia (FTD), and Parkinson’s disease (PD), non-invasive diagnostic tools are urgently needed.

**Methods:**

Plasma samples were collected from 200 individuals, divided into three groups, including young (20–40 years), middle-aged (41–60 years), and elderly (> 60 years). Exosomes were isolated, followed by small RNA sequencing (sRNA-seq) to identify differentially expressed miRNAs, and differentially expressed miRNAs related to autophagy were validated using quantitative real-time PCR (qRT-PCR). Spearman correlation analysis was performed to assess the relationship between autophagy-related miRNAs and brain aging biomarkers. Receiver operating characteristic (ROC) curve analysis was conducted to evaluate the diagnostic performance.

**Results:**

Nine autophagy-related miRNAs were identified and validated as significantly upregulated in plasma exosomal from elderly, including hsa-miR-2110, hsa-miR-18a-3p, hsa-miR-766-3p, hsa-miR-4446-3p, hsa-miR-4667-5p, hsa-miR-4433b-3p, hsa-miR-146a-5p, hsa-miR-423-5p, and novel_260. These miRNAs were validated by qRT-PCR. Correlation analysis showed that several of these miRNAs, such as hsa-miR-2110 and hsa-miR-766-3p, were strongly correlated with NfL (*r* = 0.68, *p* = 0.002), Aβ42 (*r* = 0.62, *p* = 0.004), and p-Tau181 (*r* = 0.55, *p* = 0.008). ROC curve analysis showed that combining these miRNAs with NfL resulted in an area under the curve (AUC) of 0.92, outperforming NfL alone (AUC = 0.85) and miRNAs alone (AUC = 0.84). Further subgroup analysis revealed that multiple miRNAs, such as miR-2110, miR-4446-3p, and novel_260, achieved high AUCs (>0.83) in distinguishing middle-aged adults (41–60 years) from older adults (>60 years), supporting their potential utility for early detection of age-associated neurodegeneration.

**Conclusion:**

This study identifies a set of autophagy-related miRNAs as promising biomarkers for brain aging. The combination of these miRNAs with traditional biomarkers offers a non-invasive and highly sensitive method for early detection of brain aging, providing significant potential to enhance diagnostic accuracy in neurodegenerative diseases.

## 1 Introduction

Aging is an inevitable biological process that is associated with progressive deterioration of various physiological functions, particularly in the brain. Age-related cognitive decline and neurodegenerative diseases, including Alzheimer’s disease (AD), Parkinson’s disease (PD) and Frontotemporal dementia (FTD) (Hou et al., 2019; Trist et al., 2019; Neylan and Miller, 2023). These disorders represent major healthcare challenges due to their progressive nature, lack of curative treatment, and significant socioeconomic burden. While the biological mechanisms underlying brain aging are complex, one central process that has emerged as crucial in the aging brain is autophagy, a cellular homeostasis mechanism responsible for the degradation and recycling of damaged proteins and organelles (Berglund et al., 2024). Although autophagy plays an essential role in maintaining cellular function by removing dysfunctional cellular components, its dysregulation with age is thought to contribute to neuronal dysfunction, synaptic loss, and accumulation of toxic protein aggregates, all of which are hallmarks of neurodegenerative diseases (López-Otín et al., 2023). Early detection of age-related neuronal dysfunction is critical for intervention, yet existing biomarkers—such as amyloid-*β* (Aβ), tau, and neurofilament light chain (NfL)—often become detectable only in later disease stages and require invasive procedures such as lumbar puncture or expensive imaging techniques (Zhang Z. et al., 2021; Lee et al., 2022).

Understanding the molecular mechanisms of brain aging, particularly those involving autophagy, is crucial for identifying novel biomarkers and therapeutic targets for early diagnosis and intervention in age-related neurodegeneration (Menzies et al., 2017). While much has been learned about the role of autophagy in maintaining neuronal health, there remains a significant gap in our ability to detect early changes in brain aging through biomarkers that are both sensitive and specific. The development of such biomarkers could greatly enhance our ability to diagnose neurodegenerative diseases at earlier, more treatable stages.

With aging being the greatest risk factor for neurodegenerative diseases, there is an increasing demand for accessible, non-invasive biomarkers to detect early molecular changes in the brain. Blood-based biomarkers, particularly plasma-derived exosomal miRNAs, have emerged as attractive tools due to their stability, ease of collection, and potential to detect brain-specific pathological processes (Rani et al., 2017; Wang et al., 2020). Exosomes, which are small extracellular vesicles (EVs) secreted by cells, have been shown to play a pivotal role in intercellular communication (Mathieu et al., 2021). They carry bioactive molecules, including miRNAs, proteins, and lipids, that reflect the physiological and pathological states of the cells from which they originate (Song et al., 2019). Because exosomes are present in various biological fluids, including blood, they offer an accessible and non-invasive means of monitoring brain health. To date, miRNAs, a group of short non-coding RNAs that regulate gene expression, have emerged as critical regulators of many biological processes, including stress responses, inflammation, and autophagy. Dysregulation of miRNA expression is associated with aging and neurodegenerative diseases. Notably, several exosmal miRNAs, such as miR-376a-3p, miR-15a-5p, miR-125b-5p, and miR-483-5p, have been reported to change with aging and in diseases such as AD, PD, and FTD (Zhou et al., 2021; Bao et al., 2024; Liu et al., 2023a). Compared to CSF or brain tissue sampling, exosomal miRNAs from blood offer a practical solution for routine population-level screening, longitudinal monitoring, and early diagnosis of cognitive decline. More, importantly, certain miRNAs involved in autophagy regulation have shown promise as non-invasive biomarkers for brain aging and neurodegeneration (Kou et al., 2020).

Despite the promising potential of exosomal miRNAs as biomarkers for brain aging, several key challenges remain. To date, most studies have focused on animal models or postmortem brain tissue, limiting the direct applicability of these findings to human aging. Although traditional biomarkers, such as neurofilament light chain (NfL), Aβ42, and phosphorylated tau (p-Tau181), have been valuable in monitoring disease progression, their ability to detect early changes in brain aging and distinguish between normal aging and neurodegenerative disease is limited (Higgins-Chen et al., 2021; Basisty et al., 2020). Moreover, the sensitivity and specificity of these biomarkers remain suboptimal, especially in the early stages of neurodegenerative diseases when therapeutic intervention may be most effective (Blennow and Zetterberg, 2018; Milà-Alomà et al., 2020).

This study aims to investigate the potential of plasma exosomal autophagy-related miRNAs as a novel, minimally invasive biomarker panel for brain aging. The proposed approach not only improves accessibility of screening, but also helps stratify risk for multiple age-related neurodegenerative conditions, including AD, PD, and FTD. Through the integration of cutting-edge techniques, such as small RNA sequencing (sRNA-seq) and quantitative real-time PCR (qRT-PCR) validation, this study provides new insights into the molecular mechanisms underlying brain aging and offers novel, non-invasive biomarkers for early diagnosis and monitoring of neurodegenerative diseases. By investigating the role of autophagy-related miRNAs in brain aging, this study aims to contribute to the growing body of knowledge on the molecular mechanisms of aging and provide a strong experimental foundation for developing effective diagnostic strategies for neurodegenerative diseases.

## 2 Materials and methods

### 2.1 Clinical participants

The current study was conducted on a cohort of 200 patients from Shandong Provincial Hospital between June 2022 and September 2023. *A priori* power analysis was conducted using G*Power 3.1 software to determine the minimum required sample size. Hsa-miR-2110 was selected for sample size estimation because this miRNA showed the most robust and consistent age-related increase among the nine identified miRNAs, thus representing a conservative and statistically sound example for effect size calculation. Based on the preliminary group means and standard deviations of plasma exosomal miR-2110, an effect size of Cohen’s *f* = 0.4 was estimated. Using *α* = 0.05 and statistical power = 0.9, the minimum total sample size needed to detect statistically significant differences among three age groups was calculated to be 159. Our study included 200 participants, which met the required sample size for sufficient power. A visual representation of the power calculation was shown in Supplementary Figure S1. The patients were classified into three age groups: Group I (*n* = 62) for young adults (ages 20–40 years), Group II (*n* = 67) for middle-aged adults (ages 41–60 years), and Group III (*n* = 71) for senior adults (age > 60 years). The proportion of male was 43.5, 59.7 and 50.7% in these three groups, respectively. To avoid potential bias due to the cognitive effects of COVID-19, individuals with a known history of COVID-19 infection or related clinical symptoms were excluded from the study. Other exclusion criteria included cancer, HIV, any form of brain illness, or a positive test for hepatitis B or C surface antigen antibodies. Demographic details of the participants were provided in Table 1.

**Table 1:** Baseline characteristics of participants in three age groups.

Characteristics	Young (20–40 years old; *n* = 62)	Middle-aged (41–60 years old; *n* = 67)	Elderly (> 60 years old; *n* = 71)	*p*-value
Age (year)	30.7 ± 6.2	54.5 ± 6.9	76.9 ± 6.1	< 0.001
Male: *n* (%)	27 (43.5%)	40 (59.7%)	36 (50.7%)	0.183
SBP (mm Hg)	123.3 ± 15.6	130.5 ± 17.0	141.0 ± 21.1	< 0.001
DBP (mm Hg)	82.7 ± 10.5	84.2 ± 12.2	81.2 ± 11.3	0.315
Heart rate (bpm)	81.7 ± 13.0	79.0 ± 11.4	77.5 ± 15.0	0.195
Current smoker: *n* (%)	13 (21.0%)	22 (32.8%)	20 (28.2%)	0.317
Current alcohol drinker: n (%)	19 (30.6%)	22 (32.8%)	19 (26.8%)	0.732
Diabetes mellitus: *n* (%)	2 (3.2%)	18 (26.9%)	17 (23.9%)	< 0.001
Coronary heart disease: *n* (%)	14 (22.6%)	32 (47.8%)	38 (53.5%)	< 0.001
Hypertension: *n* (%)	8 (12.9%)	25 (37.3%)	46 (64.8%)	< 0.001
Platelets (× 10^9^/L)	255.3 ± 53.6	229.6 ± 55.0	235.7 ± 78.3	0.061
HGB (g /L)	133.0 ± 19.1	136.9 ± 16.5	125.0 ± 16.7	< 0.001
RBC (× 10^12^/L)	4.6 ± 0.5	4.5 ± 0.5	4.1 ± 0.5	< 0.001
WBC (× 10^9^/L)	6.4 ± 2.2	6.1 ± 1.8	6.2 ± 1.7	0.571
LYMPH% (%)	33.2 ± 8.5	31.4 ± 7.9	29.0 ± 9.2	0.02
LYMPH (× 10^9^/L)	2.1 ± 0.6	1.8 ± 0.5	1.7 ± 0.5	0.001
NEUT% (%)	58.1 ± 9.5	59.5 ± 8.7	60.9 ± 10.0	0.243
NEUT (× 10^9^/L)	3.9 ± 1.9	3.7 ± 1.6	3.9 ± 1.6	0.836
N/L ratio	2.0 ± 1.3	2.2 ± 1.3	2.6 ± 2.0	0.089
P/L ratio	131.0 ± 40.2	133.5 ± 48.7	155.3 ± 93.0	0.064
HDL-C (mmol/L)	1.4 ± 0.4	1.2 ± 0.3	1.3 ± 0.3	0.023
LDL-C (mmol/L)	2.7 ± 0.7	3.1 ± 0.9	2.8 ± 0.9	0.021
CHOL (mmol/L)	4.4 ± 1.0	4.8 ± 1.1	4.5 ± 1.2	0.108
TG (mmol/L)	1.2 ± 0.7	1.5 ± 0.7	1.3 ± 0.6	0.11
APOB (g/L)	0.8 ± 0.3	1.0 ± 0.3	0.9 ± 0.3	0.076
APOA1 (g/L)	1.2 ± 0.3	1.2 ± 0.2	1.2 ± 0.2	0.167
ALB (g/L)	45.2 ± 6.4	42.6 ± 6.4	40.9 ± 7.0	0.001
URIC (μmol/L)	331.2 ± 95.9	324.1 ± 89.7	313.4 ± 83.0	0.509
UREA (mmol/L)	4.8 ± 1.3	6.0 ± 1.5	5.9 ± 1.7	< 0.001
HCY (μmol/L)	14.0 ± 7.9	13.7 ± 6.7	16.7 ± 8.8	< 0.001
GLU (mmol/L)	5.1 ± 1.0	5.6 ± 1.5	5.8 ± 1.7	0.021
AST (U/L)	26.9 ± 36.7	27.5 ± 17.9	23.5 ± 9.3	0.166
ALT (U/L)	29.0 ± 34.0	29.1 ± 23.9	20.5 ± 12.2	0.034
GGT (U/L)	34.5 ± 43.3	28.9 ± 18.8	28.8 ± 17.1	0.78
ALP (U/L)	70.5 ± 20.4	73.9 ± 21.4	78.6 ± 25.8	0.118
GLDH (U/L)	7.6 ± 25.7	5.0 ± 4.5	5.3 ± 7.1	0.389
CREA (μmol/L)	70.8 ± 69.5	63.1 ± 14.5	66.5 ± 18.4	0.571
eGFR	120.7 ± 13.1	106.8 ± 14.9	85.9 ± 16.2	< 0.001

Continuous variables are reported as either median, interquartile (25th to 75th percentiles), or mean ± standard deviation (SD). Significant differences between groups are examined using the Kruskal-Wallis H test and one-way ANOVA. The patients are divided into three groups according to age: young group 20–40 years old, middle-aged group 45–65 years old, and elderly group > 65 years old. SBP, systolic blood pressure; DBP, diastolic blood pressure; HGB, hemoglobin; RBC, red blood cell; WBC, white blood cell; LYMPH%, the percentage of lymphocyte; LYMPH, lymphocyte; NEUT%, the percentage of neutrophil; NEUT, neutrophil; N/L ratio, neutrophil to lymphocyte ratio; P/L ratio, platelets to lymphocyte ratio; HDL-C, high-density lipoprotein cholesterol; LDL-C, low-density lipoprotein cholesterol; CHOL, cholesterol; TG, triglyceride; APOB, apolipoprotein-B; APOA1, apolipoprotein-A1; ALB, albumin; URIC, uric acid; HCY, homocysteine; GLU, glucose; AST, aspartate aminotransferase; ALT, alanine aminotransferase; GGT, gamma-glutamyl transpeptidase; ALP, alkaline phosphatase; GLDH, glutamic acid dehydrogenase; CREA, creatinine; eGFR, glomerular filtration.

This study was approved by the Ethics Committee of the Provincial Hospital affiliated with Shandong First Medical University for Biomedical Research Involving Human Beings (NSFC No. 2022-511). The investigation was conducted in accordance with the ethical principles outlined in the Declaration of Helsinki, and written informed consent was obtained from all participants prior to their involvement in the study.

### 2.2 Plasma exosome extraction and characterization

Venous blood sample was collected from the patient on the same day of administration to the hospital and left at room temperature for 1 h before being centrifuged at 3000 rpm and 4°C for 20 min. Plasma samples were aliquoted into portions of 500 μL each and promptly kept at −80°C for subsequent EV isolation and related measurements. Plasma exosome purification and exosome RNA extraction from 500 μL of plasma were performed by using the Total Exosome Isolation Kit (catalog no. UR52151, Umibio Biotechnology, China). In brief, to remove plasma proteins, a total of 500 μL of plasma was melted at 4°C and then combined with 400 μL of Umibio exosome precipitation solution A. Then, 120 μL of solution B was added, and the mixture was centrifuged for 15 min at 12,000 rpm/min. After centrifugation, the sediments were collected and resuspended in PBS buffer. After centrifugation at 12000 × g for 2 min, supernatant was transferred to an exosome purification filter (EPF) column, then centrifuged for 10 min at 4°C. The exosomes in the collection tube were then collected and stored at 4°C or −80°C as needed.

### 2.3 Transmission electron microscopy analysis

Plasma exosomes suspended in particle-free PBS (0.02 μm filtered) were placed on a carbon-coated copper grid (200 mesh) and incubated at room temperature for 5 min. Excess liquid was then blotted with absorbent tissue. Uranyl acetate was added to the grid and allowed to stain for 10 s. Remaining stain was gently removed with filter paper, and the grid was air-dried for 30 min. The prepared exosome samples were then visualized using transmission electron microscopy (TEM; Hitachi H7700, Japan).

### 2.4 Nanoparticle tracking analysis

Plasma exosome samples were diluted in 500 μL of particle-free PBS (0.02 μm filtered) and were subjected to nanoparticle tracking analysis with the NanoSight NS300 instrument (Malvern Instruments Ltd., Malvern, UK) to determine the hydrodynamic diameter distribution and concentration of exosomes. Data were recorded using NTA 3.4 Build 3.4.4 software with camera level set to 11 and detection threshold at 5.

### 2.5 Western blot analysis

EV protein CD63, ALIX, and GM130 were identified through Western blotting. Total proteins from EV were extracted by using RIPA lysis buffer (Solarbio, China). The concentration of the proteins was quantified by using the BCA Protein Analysis kit (Beyotime, Shanghai, China) according to the manufacturer’s instructions. Then, the lysates were electrophoresed using SDS-PAGE in 10% Bis-Tris gel (Thermo Fisher Scientific, USA) and transferred onto PVDF membranes. After being blocked, the membranes were incubated with primary antibody against anti-GM130 (ab52649, Abcam, USA), anti-ALIX (ab186429, Abcam, USA), and anti-CD63 (A5271, ABclonal, China) overnight at 4°C. The secondary antibodies were incubated for 1 h at room temperature. Finally, the protein bands were treated using the Immobilon Western HRP substrate Luminol reagent (Millipore, USA) and imaged using facility of Tanon (Shanghai, China). ImageJ software (NIH, USA) was used to calculate the optical density of immunoblots.

### 2.6 Measurement of brain aging biomarkers

To assess brain aging biomarkers, frozen plasma samples were thawed at 4°C and centrifuged at 10,000 × g for 10 min to eliminate cell debris. The plasma was then divided into 75 μL aliquots, and biomarker levels were measured using a Single-Molecule Analyzer and associated assay kits (AXL-2000, Lychix, Suzhou, China).

### 2.7 Small RNA-sequencing analysis

Small RNA sequencing (sRNA-seq) analysis was used to screen the potential plasma exosomal miRNAs for brain aging diagnosis. After the candidates were selected, qRT-PCR was further performed in large samples to determine whether the candidates could be used as ideal biomarkers. Thus, six senior patients (age ≥ 80 years old) and six young patients (age ≤ 30 years old) were randomly selected for the sRNA-seq analysis. The characteristics of the twelve patients were shown in Table 2. The sRNA-seq analysis was performed using the Multiplex Small RNA Library Prep Set for Illumina® (San Diego, CA, USA) at the Novegene Bioinformatics Technology Co., Ltd. (Beijing, China), according to the manufacturer’s instructions. For annotating exosomal sRNAs, the sRNAs were filtered from clean reads based on the range of read length. The sRNA tags were mapped and matched to genome version CRCh38 by the short read aligners named by Bowtie. For identifying the known miRNAs, the sRNAs were mapped to miRBase20.0. Novel miRNAs were predicted using *miREvo* and *miRDeep2*, both of which integrated multiple features, including precursor miRNA secondary structure, Dicer cleavage patterns, minimum free energy, and base-pairing properties of the RNA hairpin structure. Differentially expressed miRNAs between senior patients and young donors were performed using the DESeq2 R package. To control for multiple testing, the Benjamini–Hochberg method was applied to calculate the false discovery rate (FDR). The log_2_(fold change) > 1 and FDR (adjusted *p* value) < 0.05 were set as the thresholds for distinguishing differentially expressed miRNAs.

**Table 2:** Target primer sequences of miRNAs.

miRNA	RNA sequence	Primer	GC content
hsa-miR-2110	UUGGGGAAACGGCCGCUGAGUG	GAAACGGCCGCTGAGTGAAA	55%
has-miR-18a-3p	ACUGCCCUAAGUGCUCCUUCUGG	ACTGCCCTAAGTGCTCCTTCT	52%
hsa-miR-766-3p	ACUCCAGCCCCACAGCCUCAGC	GCCCCACAGCCTCAGCAAAA	60%
hsa-miR-4446-3p	CAGGGCUGGCAGUGACAUGGGU	GCTGGCAGTGACATGGGTAAA	52%
hsa-miR-4667-5p	ACUGGGGAGCAGAAGGAGAACC	ACTGGGGAGCAGAAGGAGAA	55%
hsa-miR-4433b-3p	CAGGAGUGGGGGGUGGGACGU	AGTGGGGGGTGGGACGTAAA	60%
hsa-146a-5p	UGAGAACUGAAUUCCAUGGGUU	GGTGAGAACTGAATTCCATGGG	50%
hsa-miR-423-5p	UCUUGGAGUAGGUCAUUGGGUGG	CTTGGAGTAGGTCATTGGGTGG	55%
novel_260	AGGGGCGCUCGUGCUUGAGC	CGCTCGTGCTTGAGCAAA	56%

### 2.8 Exosomal RNA isolation and quantitative real-time PCR

Total RNA, including both mRNAs and miRNAs, was extracted from 200 μL plasma EV samples collected from the entire cohort of 200 participants using Total Exosome RNA and Protein Isolation Kit (ThermoFisher, cat4478545, Rockford, USA) and used to synthesize cDNA using commercially available miR-specific primers (for hsa-miR-2110, hsa-miR-18a-3p, hsa-miR-766-3p, hsa-miR-4446-3p, hsa-miR-4667-5p, hsa-miR-4433b-3p, hsa-miR-146a-5p, hsa-miR-423-5p, and novel_260) and endogenous control-specific (U6) reverse primers according to miRNA 1st strand cDNA synthesis kit (Applied Biosystems, Carlsbad, USA). Quantitative real-time PCR (qRT-PCR) was performed using Power SYBR Green PCR Master Mix (Applied Biosystems, Carlsbad, USA). The expression of the 9 miRNAs was normalized to the levels of U6 and analyzed for relative fold changes from the threshold cycle (Ct) values using the 2^−ΔΔCt^ method. The qRT-PCR analysis for each miRNA was repeated three times. The target sequences of the primer sets were shown in Table 3. The significance of differentially expressed miRNAs was measured based on the fold change and *p*-value/*q*-value.

**Table 3:** Readcount based on miRNA differential expression analysis.

miRNA	Elderly (>80 years old) readcount	Young (18–30 years old) readcount	log_2_(Fold Change)	*p*-value	*q*-value	significant
hsa-miR-2110	58.78092	25.49299	1.176031	0.016666	0.248369	True
hsa-miR-4433b-3p	4893.898	1556.858	1.652264	0.024497	0.299976	True
hsa-miR-4667-5p	17.48317	2.477344	2.704715	0.046667	0.371401	True
hsa-miR-18a-3p	0	4.4911	−4.82979	0.001916	0.083637	True
hsa-miR-766-3p	1.986141	9.764105	−2.26937	0.036915	0.371401	True
hsa-miR-4446-3p	107.195	34.9835	1.571621	0.03381	0.371401	True
hsa-miR-146a-5p	40011.23	17606.21	1.184328	0.030435	0.34437	True
hsa-miR-423-5p	43372.47	15750.54	1.46149	0.012792	0.234053	True
novel_260	4.920685	0	4.598652	0.043289	0.371401	True

### 2.9 Statistics

Statistical analysis was conducted using R software (The R Foundation; http://www.r-project.org/; version 3.4.3) and Empower (R) (www.empowerstates.com; X&Y Solutions Inc., Boston, MA, USA). Continuous variables were presented as either medians with interquartile ranges (25th to 75th percentiles) or means ± standard deviation (SD). Group differences were assessed via the Kruskal-Wallis H test and one-way analysis of variance (ANOVA). Spearman correlation analysis was used to assess the relationship between exosomal miRNA levels and age of patients, while multivariable linear regression analyzed the associations between exosomal miRNA levels and various brain aging biomarkers. To evaluate the diagnostic accuracy of exosomal-miRNA and plasma brain aging biomarkers, both univariate and multivariate logistic regression analyses were performed and ROC models were constructed to compare different age groups.

## 3 Results

### 3.1 Experimental workflow for identifying autophagy-related miRNAs as brain aging biomarkers

**Figure 1:**
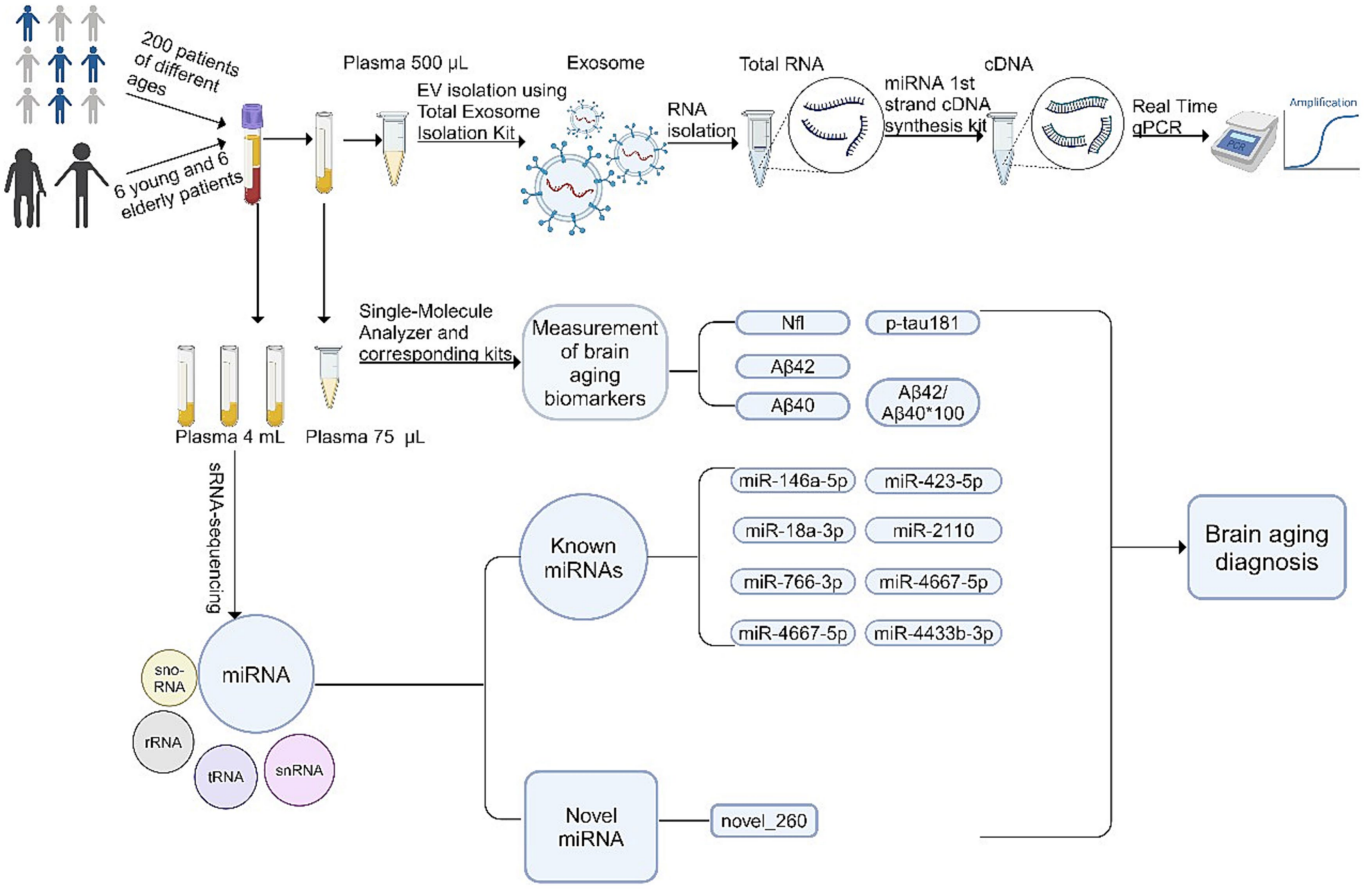
Plasma exosomal miRNAs holding significant potential for diagnosing brain aging. Based on extracellular vesicle (EV) purification, sRNA sequencing, and qRT-PCR, a total of 9 exosomal miRNAs are identified as potential biomarkers for brain aging diagnosis.

This study aimed to identify plasma exosomal miRNAs as potential biomarkers for brain aging. Figure 1 provided a comprehensive schematic of the experimental workflow. The plasma samples were collected from 200 participants, categorized into three age groups: young (20–40 years), middle-aged (41–60 years), and elderly (>60 years). After isolating exosomes from plasma, sRNA-seq was performed to identify differentially expressed miRNAs between young and elderly groups. The baseline characteristics of the participants, including key demographic and clinical variables, such as age, systolic blood pressure, and heart rate, were presented in Table 2. The results showed significant differences across the age groups (*p* < 0.05, Kruskal-Wallis H test). This stratification ensured that the analysis accounted for age-related differences in clinical measures.

Further analysis of the sRNA composition in plasma exosomes revealed notable variations between young and elderly groups (Supplementary Figures S2, S3). The results indicated a higher proportion of certain miRNAs and other sRNAs in the elderly group, suggesting aging-associated changes in RNA metabolism and function.

Differential expression analysis of plasma exosomal miRNAs was conducted using DESeq2, with multiple testing correction performed using the Benjamini–Hochberg method to control the FDR. MiRNAs with adjusted *p*-values (*q*-values) less than 0.4 were considered statistically significant for exploratory purposes. Based on these criteria, a total of 9 miRNAs were identified as significantly differentially expressed in plasma exosomes from the elderly group (> 80 years old) compared to the young group (18–30 years old), including hsa-miR-2110, hsa-miR-4433b-3p, hsa-miR-4667-5p, hsa-miR-18a-3p, hsa-miR-766-3p, hsa-miR-4446-3p, hsa-miR-146a-5p, hsa-miR-423-5p, and novel_260. It was noted that while the raw *p*-values indicated statistical significance (all < 0.05), the FDR-adjusted p-values (q-values) ranged from 0.0836 to 0.3714 (Table 4), indicating the moderate statistical significance after multiple comparisons. Therefore, these results were regarded as exploratory, warranting further validation in larger cohorts. Notably, “novel_260” was one of the top candidates, exhibiting a characteristic hairpin structure and consistent expression across multiple samples. The sequence information, expression levels, and structural visualization of “novel_260” were provided in Tables 3, 4 and Supplementary Figure S4, respectively. These miRNAs were retained for downstream analysis based on their biological relevance, consistent expression trends, and potential involvement in aging-related regulatory pathways.

**Table 4:** Differential analysis of autophagy-related miRNAs associated with brain aging based on small RNA sequencing.

Gene	miRNA
*ATG5*	hsa-miR-4446-3p, hsa-4667-5p
*RRAGA*	hsa-miR-4667-5p
*TSC1*	hsa-miR-4446-3p
*TP53INP2*	hsa-miR-4433b-3p
*SQSTM1*	hsa-miR-2110, hsa-miR-18a-3p, novel_260
*BCL2*	hsa-miR-766-3p
*CTSD*	hsa-miR-146a-5p, hsa-miR-423-5p
*TEFB*	hsa-miR-18a-3p, hsa-miR-766-3p, hsa-miR-4446-3p

### 3.2 Differential expression of miRNAs and autophagy-related pathways

**Figure 2:**
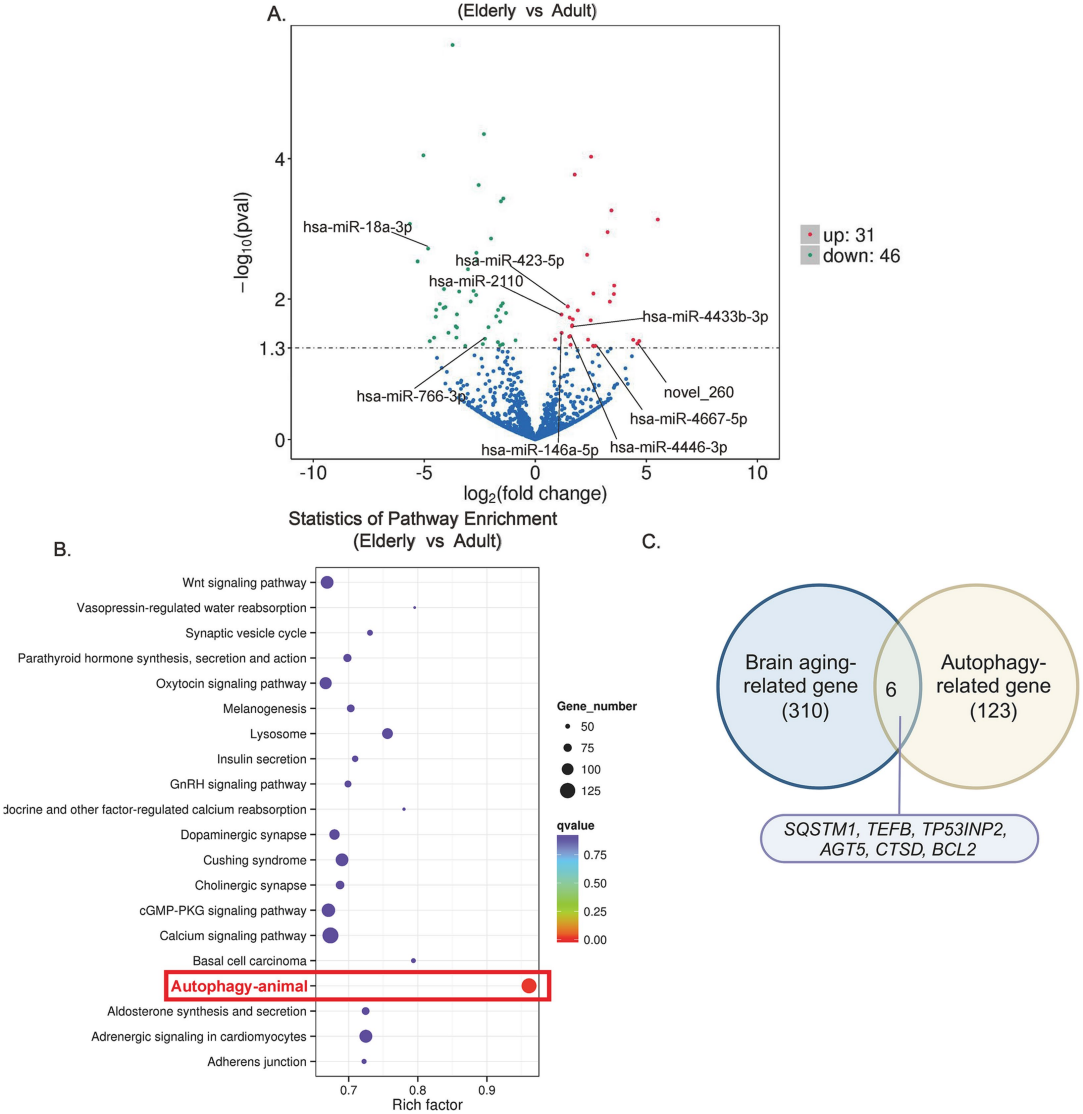
KEGG enrichment analysis based on differentially expressed genes (DEGs) of elderly individuals vs. adults. **(A)** Volcano plot illustrating differentially expressed plasma exosomal miRNAs of elderly vs. adults based on sRNA sequencing. **(B)** Scatter diagram of KEGG enrichment analysis showing that the differentially expressed miRNAs are significantly enriched in pathways related to autophagy (highlighted in a red box). **(C)** Intersecting brain aging-related genes from the AgeAnno database with autophagy-related genes identified from sRNA sequencing indicating autophagy genes associated with brain aging.

To further explore the involvement of these miRNAs in brain aging, we examined their association with key aging-related pathways. The volcano plot of the differential expression of miRNAs between elderly and young groups revealed a substantial number of upregulated miRNAs in the elderly cohort, with several linked to aging and neurodegeneration (Figure 2A).

The results of KEGG enrichment analysis showed that the differentially expressed miRNAs were significantly enriched in pathways related to autophagy, oxidative stress, and neurodegenerative diseases (Figure 2B and Supplementary Figure S5). Notably, the autophagy pathway was prominently represented, underscoring its critical role in the aging brain.

Further intersecting brain aging-related genes from the AgeAnno database with autophagy-related genes identified in sRNA-seq (Figure 2C) revealed several key autophagy-related genes, such as *Atg5, Beclin1*, and *SQSTM1*, that were strongly associated with brain aging, reinforcing the importance of autophagy in the aging pathophysiology (Table 1).

Based on integrated target prediction using databases miRDB and TargetScan, several upregulated miRNAs were identified to potentially regulate autophagy-related genes. For instance, miR-2110 and miR-18a-3p were predicted to target SQSTM1, while miR-4667-5p and miR-4446-3p may target ATG5. These predicted interactions suggested that elevated miRNA levels might contribute to autophagic dysfunction in the aging brain. However, it was noted that direct experimental validation of these interactions, such as dual-luciferase reporter assays, would be necessary to verify these findings using both *in vitro* and *in vivo* functional validations in future investigations to better elucidate the regulatory mechanisms of these miRNAs in modulating autophagy during brain aging.

### 3.3 Quality control of exosomes and RNA integrity

**Figure 3:**
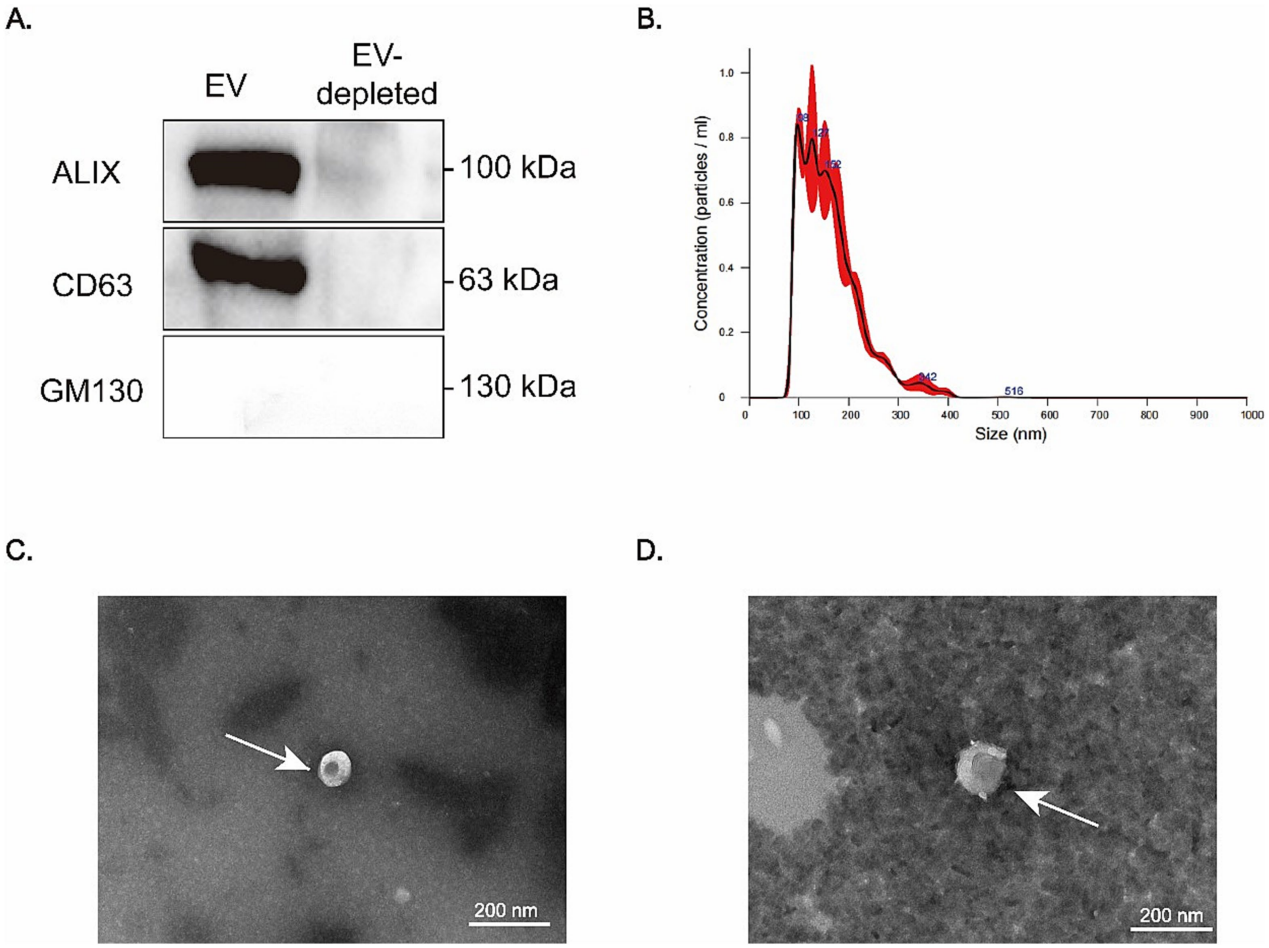
Isolation and characterization of plasma-derived extracellular vesicles (EVs). **(A)** Western blot analysis of EV markers (ALIX and CD63) and the cellular contamination marker (GM130). ALIX and CD63 are detected in the EV fraction, while GM130 is absent, confirming effective removal of cellular debris. **(B)** Nanoparticle tracking analysis (NTA) revealing that the size distribution of isolated EVs predominantly falls within the 100–300 nm range. **(C,D)** Transmission electron microscopy (TEM) images showing the characteristic cup-shaped morphology of EVs, which are indicated by white arrows.

Prior to the validation of the identified miRNAs, we ensured the integrity of the exosomal samples and RNA extracted. The results of Western blotting confirmed the presence of two exosomal markers (ALIX and CD63), and the absence of GM130, a marker for cellular contamination (Figure 3A). These results validated the purity of the isolated exosomes.

To further assess the quality of the exosomes, the results of NTA indicated that the exosomes predominantly fell within the size range of 100–300 nm (Figure 3B), consistent with the expected size for exosomes. TEM images further confirmed the characteristic cup-shaped morphology of exosomes (Figures 3C,D), verifying their structural integrity.

RNA integrity was also assessed, with high-quality RNA confirmed by the clear separation of miRNA and mRNA bands, ensuring that the RNA extracted from plasma exosomes was suitable for downstream qRT-PCR validation.

Additionally, the length distribution of sRNAs in plasma exosomes was further explored in both elderly and young groups (Supplementary Figure S3). The results revealed distinct patterns in sRNA length distributions between the two groups, suggesting variations in RNA biogenesis or stability associated with aging.

### 3.4 Validation of autophagy-related miRNAs as biomarkers for brain aging

We validated the 9 miRNAs identified (i.e., hsa-miR-2110, hsa-miR-18a-3p, hsa-miR-766-3p, hsa-miR-4446-3p, hsa-miR-4667-5p, hsa-miR-4433b-3p, hsa-miR-146a-5p, hsa-miR-423-5p, and novel_260) and brain aging biomarkers (i.e., NfL, Aβ42, and p-Tau181) in the full cohort of 200 participants using qRT-PCR analysis (Tables 5, 6). The results confirmed that these miRNAs were significantly upregulated in the elderly group, compared to the young group (*p* < 0.05, Kruskal-Wallis H test), consistent with the findings from sRNA-seq.

**Table 5:** Distribution of plasma exosomal miRNA levels in three different age groups.

Characteristics	Young (20–40 years old; *n* = 62)	Middle-aged (41–60 years old; *n* = 67)	Elderly (> 60 years old; *n* = 71)	*p-*value
hsa-miR-2110	19.8 ± 11.5	85.5 ± 120.4	186.3 ± 154.5	< 0.001
hsa-miR-18a-3p	264.7 ± 193.3	58.1 ± 97.1	27.7 ± 22.2	< 0.001
hsa-miR-766-3p	210.9 ± 193.2	61.1 ± 86.2	27.1 ± 17.2	< 0.001
hsa-miR-4446-3p	18.4 ± 10.7	96.1 ± 132.0	170.9 ± 198.5	< 0.001
hsa-miR-4667-5p	18.5 ± 11.1	78.6 ± 97.8	192.0 ± 197.2	< 0.001
hsa-miR-4433b-3p	20.8 ± 12.4	87.5 ± 122.1	184.3 ± 166.7	< 0.001
hsa-miR-146a-5p	19.1 ± 11.9	78.1 ± 104.9	158.2 ± 124.8	< 0.001
hsa-miR-423-5p	20.3 ± 11.9	85.6 ± 105.4	198.5 ± 182.4	< 0.001
novel_260	19.5 ± 10.8	72.2 ± 107.1	143.0 ± 134.2	< 0.001

Continuous variables are presented as mean ± standard deviation (SD). Statistical differences between groups are assessed using the Kruskal-Wallis H test and one-way ANOVA.

**Table 6:** Distribution of levels of plasma brain aging biomarkers in three different age groups.

Characteristics	Young (20–40 years old; *n* = 62)	Middle-aged (41–60 years old; *n* = 67)	Elderly (> 60 years old; *n* = 71)	*p*-value
NfL (pg/mL)	4.8 ± 3.6	13.9 ± 10.0	49.3 ± 18.0	< 0.001
Aβ42 (pg/mL)	5.0 ± 3.0	5.3 ± 1.6	6.7 ± 3.7	0.001
p-Tau181 (pg/mL)	2.8 ± 3.7	3.7 ± 5.5	2.9 ± 1.9	0.075
Aβ40 (pg/mL)	373.4 ± 134.9	392.4 ± 141.4	474.5 ± 162.5	< 0.001
Aβ42/Aβ40*100	1.5 ± 1.0	1.5 ± 0.6	1.6 ± 1.1	0.597

Continuous variables are presented as mean ± standard deviation (SD). Statistical differences between groups are assessed using the Kruskal-Wallis H test and one-way ANOVA.

**Figure 4:**
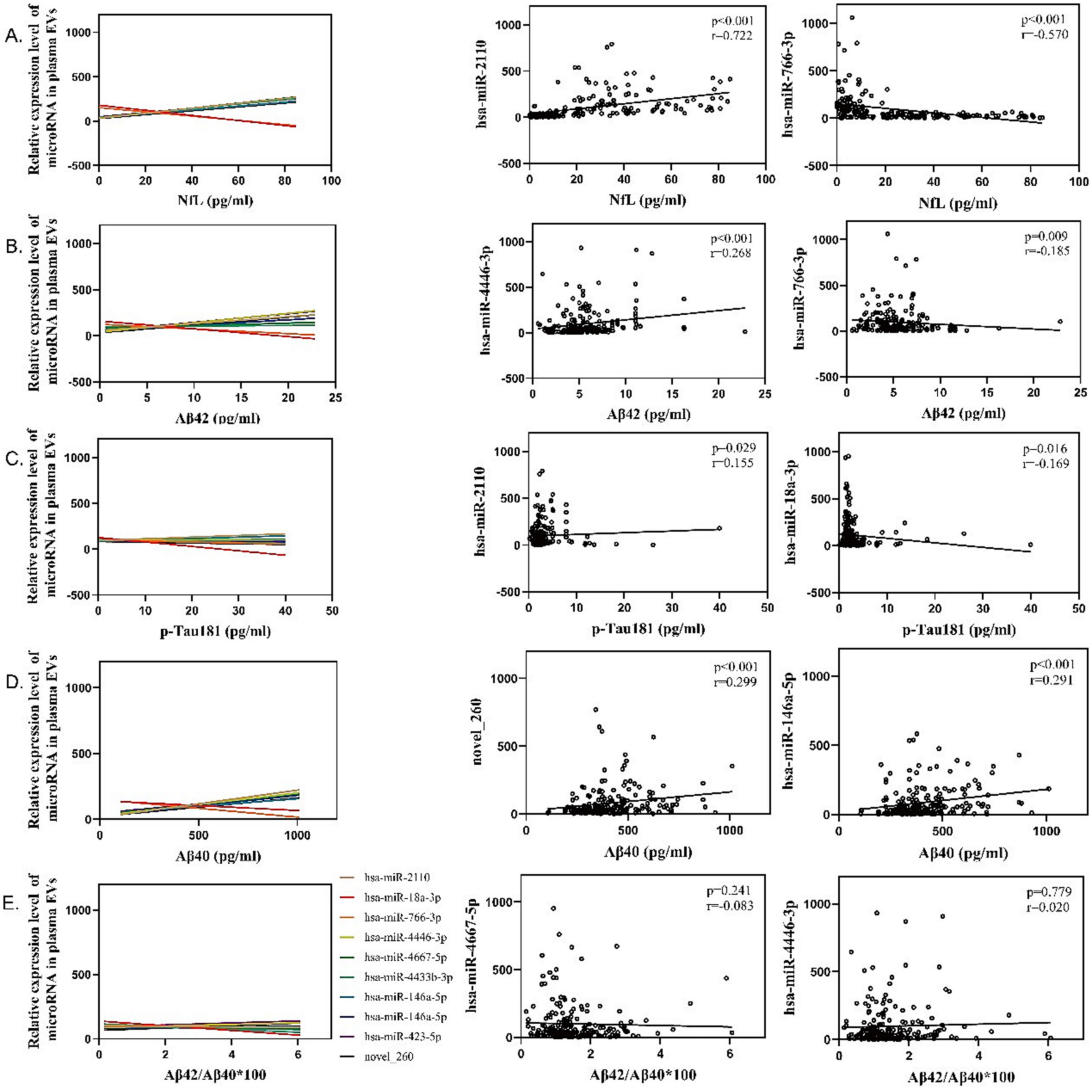
Spearman correlation analysis between plasma exosomal miRNA expression (measured in arbitrary units, AU) and plasma biomarkers related to brain aging, including **(A)** neurofilament light chain (NfL; pg./ml), **(B)** amyloid-beta 42 (A*β*42; pg./ml), **(C)** phosphorylated Tau181 (p-Tau181; pg./ml), **(D)** amyloid-beta 40 (Aβ40; pg./ml), and **(E)** the ratio of Aβ42 to Aβ40. Spearman correlation coefficient (*r*) and *p-*values are indicated for each panel.

To explore the functions of these miRNAs, we performed Spearman correlation analysis to examine their relationships with brain aging biomarkers, such as NfL, A*β*42, and p-Tau181 (Figure 4 and Supplementary Figure S5). The results revealed significantly positive correlations between miRNAs and biomarkers. For example, hsa-miR-2110 showed a strong correlation with NfL (*r* = 0.68, *p* = 0.002), while hsa-miR-18a-3p exhibited a significant correlation with A*β*42 (r = 0.62, *p* = 0.004), suggesting that these miRNAs were involved in the neurodegenerative processes associated with brain aging.

These findings underscored the potential of these autophagy-related miRNAs as biomarkers for monitoring brain aging and neurodegenerative diseases.

### 3.5 Diagnostic potential of autophagy-related miRNAs for brain aging

To further validate the above observed associations, a multivariable regression analysis was conducted to adjust for potential confounding factors (Table 7 and Supplementary Table S1). In the unadjusted model, for example, miR-18a-3p levels were significantly associated with NfL (*β* = −2.872, *p* < 0.001), Aβ42 (β = −8.421, *p* = 0.02455), and Aβ40 (β = −0.075, *p* = 0.30944) (Figures 4A–E). However, after adjusting for factors in Model B, i.e., age, diabetes, hypertension, coronary heart disease, SBP, HGB, RBC, lymphocyte count, albumin, urea, homocysteine (HCY), glucose (GLU), alanine aminotransferase (ALT), and estimated glomerular filtration rate (eGFR), only the association between miR-18a-3p, miR-766-3p, miR-4446-3p levels and NfL remained statistically significant (β = 2.332, *p* = 0.00768, β = 2.133, *p* = 0.01688, and β = 1.989, *p* = 0.04456) (Table 7 and Supplementary Table S1). These results suggested that the association between miRNAs and NfL was independent of other confounding factors, indicating the potential of miRNAs as a specific biomarker for neuronal injury.

**Table 7:** Multivariable regression analysis assessing the association between exosomal miRNA levels and the plasma brain aging biomarker NfL, with associations for other brain aging biomarkers and exosomal miRNAs provided in Supplementary Table S1.

miRNA	NfL (pg/mL)					
	Non-adjusted		Model A		Model B	
	*p*-value	β (95%CI)	*p*-value	β (95%CI)	*p*-value	β (95%CI)
hsa-miR-2110	< 0.00001	2.740 (2.023, 3.457)	0.5845	0.379 (−0.978, 1.737)	0.90984	0.093 (−1.515, 1.701)
has-miR-18a-3p	< 0.00001	−2.872 (−3.754, −1.991)	0.00009	2.963 (1.510, 4.416)	0.00768	2.332 (0.637, 4.027)
hsa-miR-766-3p	< 0.00001	−2.387 (−3.182, −1.592)	0.05821	1.398 (−0.040, 2.836)	0.01688	2.133 (0.400, 3.867)
hsa-miR-4446-3p	< 0.00001	2.783 (1.939, 3.628)	0.22297	1.021 (−0.616, 2.659)	0.04456	1.989 (0.062, 3.917)
hsa-miR-4667-5p	< 0.00001	2.814 (2.003, 3.626)	0.61887	0.394 (−1.155, 1.942)	0.67567	0.387 (−1.422, 2.196)
hsa-miR-4433b-3p	< 0.00001	2.507 (1.741, 3.273)	0.79852	−0.188 (−1.631, 1.255)	0.58353	−0.459 (−2.096, 1.179)
hsa-146a-5p-1	< 0.00001	2.229 (1.629, 2.830)	0.97206	0.020 (−1.105, 1.146)	0.5006	−0.453 (−1.767, 0.862)
hsa-miR-423-5p	< 0.00001	2.419 (1.610, 3.227)	0.26494	−0.856 (−2.356, 0.644)	0.08951	−1.536 (−3.299, 0.227)
novel_260	< 0.00001	2.167 (1.553, 2.780)	0.25684	0.687 (−0.497, 1.870)	0.69416	0.278 (−1.105, 1.661)

One variable (age) is adjusted in Model A; the list of variables adjusted in Model B includes age (Model A), diabetes, hypertension, coronary heart disease, SBP, HGB, RBC, the number of lymphocytes, the percentage of lymphocyte, neutrophil to lymphocyte ratio, ALB, UREA, HCY, GLU, ALT, and eGFR.

**Figure 5:**
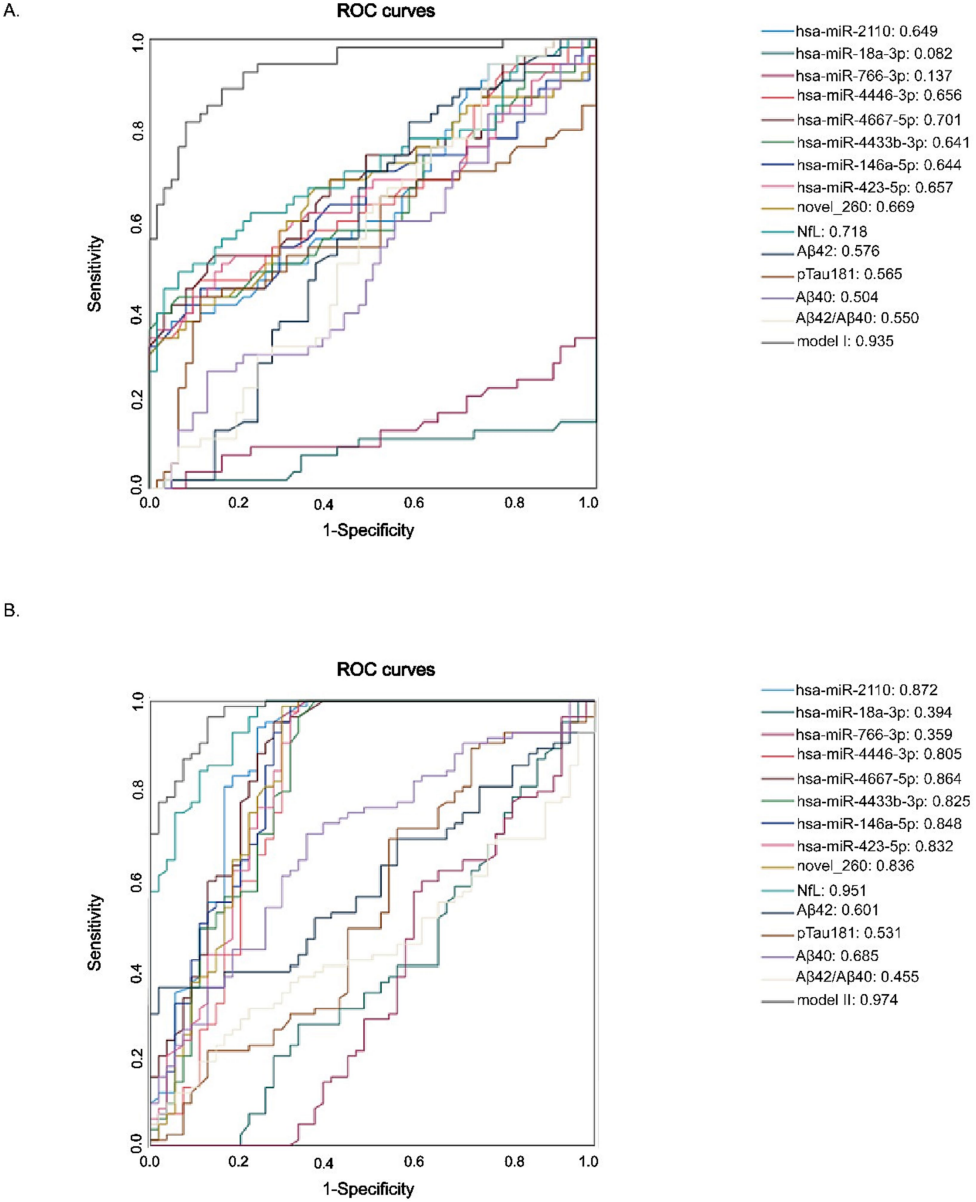
ROC curves discriminating diffenrent age groups. **(A)** Young (20–40 years) vs. Middle-aged (41–60 years). **(B)** Middle-aged (41–60 years) vs. Elderly (>60 years).

To evaluate the diagnostic potential of the identified miRNAs, participants were divided into three age groups: young (20–40 years), middle-aged (41–60 years), elderly (>60 years). Diagnostic models were established for ROC analysis: Model I compared the young group (20–40 years) with the middle-aged group (41–60 years); and Model II compared the middle-aged group (41–60 years) with the elderly group (>60 years) (Figures 5A,B). Both models incorporated 9 autophagy-related miRNAs combined with NfL, Aβ42, Aβ40, Aβ42/Aβ40 and p-Tau181. The results revealed a clear age-associated increase in diagnostic accuracy. Specifically, Model I yielded an AUC of 0.935 (95% CI: 0.891–0.979), and Model II achieved a higher AUC of 0.974 (95% CI: 0.953–0.995), suggesting enhanced discriminative power as age increased.

To further assess the discriminative capacity of all nine candidate miRNAs and classical biomarkers between the elderly and the middle-aged groups, we conducted additional ROC analysis comparing these two age groups (Table 8). These results showed that several miRNAs demonstrated strong discriminative ability, including: miR-2110 (AUC = 0.872, 95% CI: 0.802–0.943), miR-4446-3p (AUC = 0.805, 95% CI: 0.716–0.894), miR-4667-5p (AUC = 0.864, 95% CI: 0.794–0.934), miR-4433b-3p (AUC = 0.825, 95% CI: 0.7744–0.9905), miR-146a-5p (AUC = 0.848, 95% CI: 0.772–0.9923), miR-423-5p (AUC = 0.832, 95% CI: 0.752–0.912), novel_260 (AUC = 0.836, 95% CI: 0.756–0.917), and others, such as miR-4446-3p, miR-4433b-3p, and miR-423-5p (AUCs > 0.80). Importantly, many of these miRNAs showed high sensitivity, such as 98.8% for novel_260 and 94% for miR-2110, whereas the specificity level was comparatively lower, suggesting their potential utility in early detection prior to the onset of clinical symptoms. Among classical biomarkers, NfL exhibited the strongest diagnostic power (AUC = 0.951), whereas Aβ42, Aβ40, and p-Tau181 performed poorly (AUCs < 0.7) between these two age groups.

**Table 8:** ROC curves analysis to stratify different age groups using miRNA, plasma brain aging biomarkers, and euro-cognitive evaluations.

miRNA	AUC	95% CI	Cut-off	Sensitivity	Specificity
Young vs Middle-aged					
hsa-miR-2110	0.649	0.547–0.751	38.06571248	0.37	0.952
hsa-miR-18a-3p	0.082	0.022–0.142	0.512099961	1	0
hsa-miR766-3p	0.137	0.064–0.211	2.024199922	1	0
hsa-miR4446-3p	0.656	0.553–0.759	31.84148087	0.463	0.887
hsa-miR4667-5p	0.701	0.604–0.799	32.88314634	0.519	0.855
hsa-miR4433b-3p	0.641	0.536–0.746	43.4984449	0.389	0.984
hsa-miR146a-5p	0.644	0.539–0.75	35.23627163	0.444	0.887
hsa-miR423-5p	0.657	0.552–0.761	31.34689617	0.5	0.839
novel_260	0.669	0.567–0.771	40.25831936	0.333	0.968
NfL	0.718	0.621–0.815	10.302	0.481	0.935
Ab42	0.576	0.471–0.681	4.086	0.815	0.419
p-Tau181	0.565	0.453–0.677	2.840330465	0.426	0.887
Aβ40	0.504	0.398–0.611	467.146	0.259	0.871
Aβ42/Aβ40*100	0.55	0.445–0.655	0.844526114	0.944	0.242
Model I	0.935	0.891–0.979	0.526711	0.815	0.919
Middle-aged vs elderly					
hsa-miR-2110	0.872	0.802–0.943	44.19480203	0.94	0.759
hsa-miR-18a-3p	0.394	0.295–0.494	3.232914893	1	0.037
hsa-miR766-3p	0.359	0.257–0.46	4.874124901	0.964	0.074
hsa-miR4446-3p	0.805	0.716–0.894	38.78564566	0.964	0.704
hsa-miR4667-5p	0.864	0.794–0.934	43.37847256	0.952	0.722
hsa-miR4433b-3p	0.825	0.744–0.905	47.11493291	0.964	0.667
hsa-miR146a-5p	0.848	0.772–0.923	41.90009806	0.988	0.685
hsa-miR423-5p	0.832	0.752–0.912	45.26882045	0.988	0.667
novel_260	0.836	0.756–0.917	41.01593138	0.988	0.704
NfL	0.951	0.918–0.983	19.12062606	0.988	0.759
Ab42	0.601	0.507–0.694	7.144	0.357	0.981
p-Tau181	0.531	0.429–0.634	1.404	0.893	0.278
Aβ40	0.685	0.594–0.775	374.424	0.702	0.648
Aβ42/Aβ40*100	0.455	0.359–0.55	1.79378226	0.31	0.778
Model II	0.974	0.953–0.995	0.3388259	0.964	0.87

Together, these findings demonstrate that autophagy-related exosomal miRNAs—either alone or combined with NfL—possess superior diagnostic capability in distinguishing middle-aged adults from older individuals. This supports their potential as non-invasive early indicators of neurodegenerative risk, particularly the shift from later-life vulnerability to neurodegeneration.

## 4 Discussion

In this study, we identified a total of 9 autophagy-related miRNAs in plasma exosomes that were significantly upregulated in elderly individuals, providing new insights into the role of miRNAs in brain aging and neurodegeneration. Our findings suggest that dysregulated autophagy is central to brain aging and underscore the potential of exosomal miRNAs as biomarkers for the early detection of brain aging associated with neurodegeneration. By focusing on autophagy-related miRNAs, this work extends previous research by providing a novel approach to diagnosing brain aging and offering a non-invasive, sensitive method for monitoring neurodegenerative diseases at early stages.

The identified exosomal miRNAs, including hsa-miR-2110, hsa-miR-18a-3p, hsa-miR-766-3p, hsa-miR-4446-3p, hsa-miR-4667-5p, hsa-miR-4433b-3p, hsa-miR-146a-5p, hsa-miR-423-5p, and novel_260, were involved in regulating key autophagy genes, such as *Atg5*, *TFEB*, and *SQSTM1*, all of which play crucial roles in maintaining cellular autophagy homeostasis. These miRNAs were significantly upregulated in plasma exosomes of elderly individuals, suggesting that autophagic pathway is dysregulated in the aging brain. The upregulation of these miRNAs likely reflects an adaptive response to the accumulation of damaged proteins and organelles, a hallmark of aging and neurodegenerative diseases. Detecting these miRNAs in plasma exosomes offers a valuable opportunity for the early identification of brain aging, as they may act as sensitive indicators of molecular changes preceding the onset of clinical neurodegenerative symptoms.

Exosomal miRNAs offer several advantages over traditional biomarkers, such as NfL, Aβ42, and p-Tau181, which are typically elevated only in the later stages of disease progression (Piehl et al., 2022; Brickman et al., 2021; Khalil et al., 2024). Although these biomarkers are invaluable for tracking neurodegenerative diseases once they have progressed, their sensitivity is often limited in early-stage aging and neurodegeneration. In contrast, exosomal miRNAs, as molecular regulators of cellular processes, offer an early window into brain aging, allowing for the detection of molecular changes that occur prior to the onset of clinical symptoms (Toyama et al., 2019; Zhang N. et al., 2021). This makes them ideal candidates for early diagnosis of aging and neurodegeneration. Importantly, our study identifies a novel diagnostic strategy by integrating autophagy-related plasma miRNAs with NfL (Model I and Model II), a combination not previously explored in the context of brain aging. This integrated model not only enhances diagnostic sensitivity and specificity, but also represents a significant advancement over the use of traditional biomarkers alone.

Our findings are consistent with previous research, demonstrating that miRNAs are important regulators of aging and neurodegeneration (Juźwik et al., 2019; Wallach et al., 2021). For instance, miR-34a, miR-155, and miR-146a are well-established as senescence-associated miRNAs that regulate inflammation, cellular stress responses, and autophagy (Winkler et al., 2023; Zhao et al., 2019). Although miRNAs involved in inflammation and oxidative stress have been extensively studied in age-related diseases, such as AD, our study uniquely highlights a distinct panel of autophagy-related miRNAs associated with brain aging. Notably, this is the first report demonstrating the upregulation of several miRNAs—including hsa-miR-2110, hsa-miR-766-3p, hsa-miR-4667-5p, and novel_260—in plasma exosomes from elderly individuals, with an explicit focus on their potential regulation of autophagy-related genes. Unlike previous studies that emphasized inflammation-related miRNAs, we present a novel autophagy-centered biomarker panel with diagnostic relevance. This novel insight underscores the innovation of our study in identifying early diagnostic markers associated with autophagy dysregulation during brain aging and offers novel insights into the molecular mechanisms underlying neurodegeneration.

The upregulation of these miRNAs suggests a disruption in autophagic processes that could contribute to the accumulation of neurotoxic aggregates, such as Aβ and tau, which are known to impair neuronal function and lead to cognitive decline. Autophagy is essential for maintaining the quality of neuronal cells by removing damaged proteins and organelles (Fleming et al., 2022; Zhang X. et al., 2021). Dysregulation of this process leads to the accumulation of protein aggregates, a hallmark of neurodegenerative diseases. By identifying miRNAs that regulate autophagy, we could not only identify new biomarkers for early detection but also highlight potential therapeutic targets for modulating autophagy to slow down or prevent neurodegeneration (Heckmann et al., 2019; Eshraghi et al., 2021; Tang et al., 2024).

The integration of autophagy-related exosomal miRNAs (e.g., miR-2110, miR-766-3p) with traditional biomarkers such as NfL demonstrates significant promise for large-scale community screening of brain aging. First, the non-invasive nature of plasma exosome collection aligns with the logistical demands of population-based studies, enabling cost-effective and repeatable sampling. Second, the enhanced diagnostic accuracy (AUC = 0.92 for the combined model) suggests that this approach could stratify individuals at risk of early neurodegeneration, even in pre-symptomatic stages. For instance, community health centers could adopt a two-step screening protocol: initial miRNA profiling via qRT-PCR followed by confirmatory NfL testing for high-risk subgroups. This strategy would reduce the need for invasive CSF sampling or expensive neuroimaging in primary care settings. Furthermore, longitudinal monitoring of these biomarkers may track therapeutic responses in clinical trials targeting autophagy pathways. However, standardization of exosome isolation protocols and establishment of age-specific reference ranges are critical next steps to translate this research into real-world applications.

While the findings revealed in our study are promising, there are limitations that need to be addressed in future research. First, the sample size was relatively small, and the cohort was homogeneous in terms of age, which may limit the generalizability of the findings. Larger and more diverse populations are necessary to confirm these miRNAs as reliable biomarkers for brain aging in different demographic and clinical settings. Second, another limitation lies in our selection of U6 as the internal control for qRT-PCR analysis of exosomal miRNAs. Although U6 has been used in several previous studies, it may not be the most stable reference gene for plasma-derived exosomes (Liu et al., 2023b; Tang et al., 2023). Evidence suggests that miR-16 and miR-26a-5p exhibits more consistent expression in this context and may serve as a more suitable internal control (Zhou et al., 2021; Wei et al., 2020; Hu et al., 2018). Future studies should include an evaluation of internal control stability, e.g., the qRT-PCR validation experiments using miR-16 or miR-26a-5p, to enhance the reliability of miRNA quantification in exosomal studies. Third, while we demonstrated significant upregulation of autophagy-related miRNAs, the precise molecular mechanisms underlying the effects of these miRNAs on autophagic activities and their contribution to brain aging remain unclear. Future studies should focus on elucidating the functions of these miRNAs in modulating autophagic processes and their direct impact on neuronal health during brain aging. Finally, while plasma exosomes are a promising source of miRNAs for diagnostic purposes, future studies should explore other biofluids, such as cerebrospinal fluid (CSF) and saliva, to determine whether they offer additional diagnostic advantages. Comparing exosomal miRNAs from different sources could further refine the approach to detecting brain aging and neurodegenerative diseases.

In conclusion, this study presents a novel panel of exosome-derived autophagy-related miRNAs associated with brain aging, including the first-time identification of several miRNA candidates. By integrating these miRNAs with the well-established biomarker NfL, we propose a new combination strategy that significantly enhances early detection performance in the treatment of neurodegenerative diseases. These findings expand the current understanding of miRNA-mediated autophagic dysregulation during brain aging and neurodegeneration. Importantly, exosomal miRNAs offer a non-invasive and sensitive diagnostic approach that may complement or even surpass traditional biomarkers in early-stage detection of neurodegenerative diseases. While further validation and mechanistic studies are warranted to support these findings and biomarker standardization, the integration of autophagy-related miRNAs with established biomarkers, such as NfL, provides a powerful strategy for the early diagnosis and potential prevention of neurodegenerative diseases.

## Data Availability

The datasets presented in this study can be found in online repositories. The names of the repository/repositories and accession number(s) can be found in the article/Supplementary material.
