# Supplementary material for: Non-invasive biomarkers for brain aging: the role of autophagy-related microRNAs in plasma exosomes
Source: Front Mol Neurosci. 2025 Jun 6;18:1588007. doi: 10.3389/fnmol.2025.1588007 (PMC12179099; doi:10.3389/fnmol.2025.1588007)
Supplement: Supplementary file 1 [file Table_1.docx]

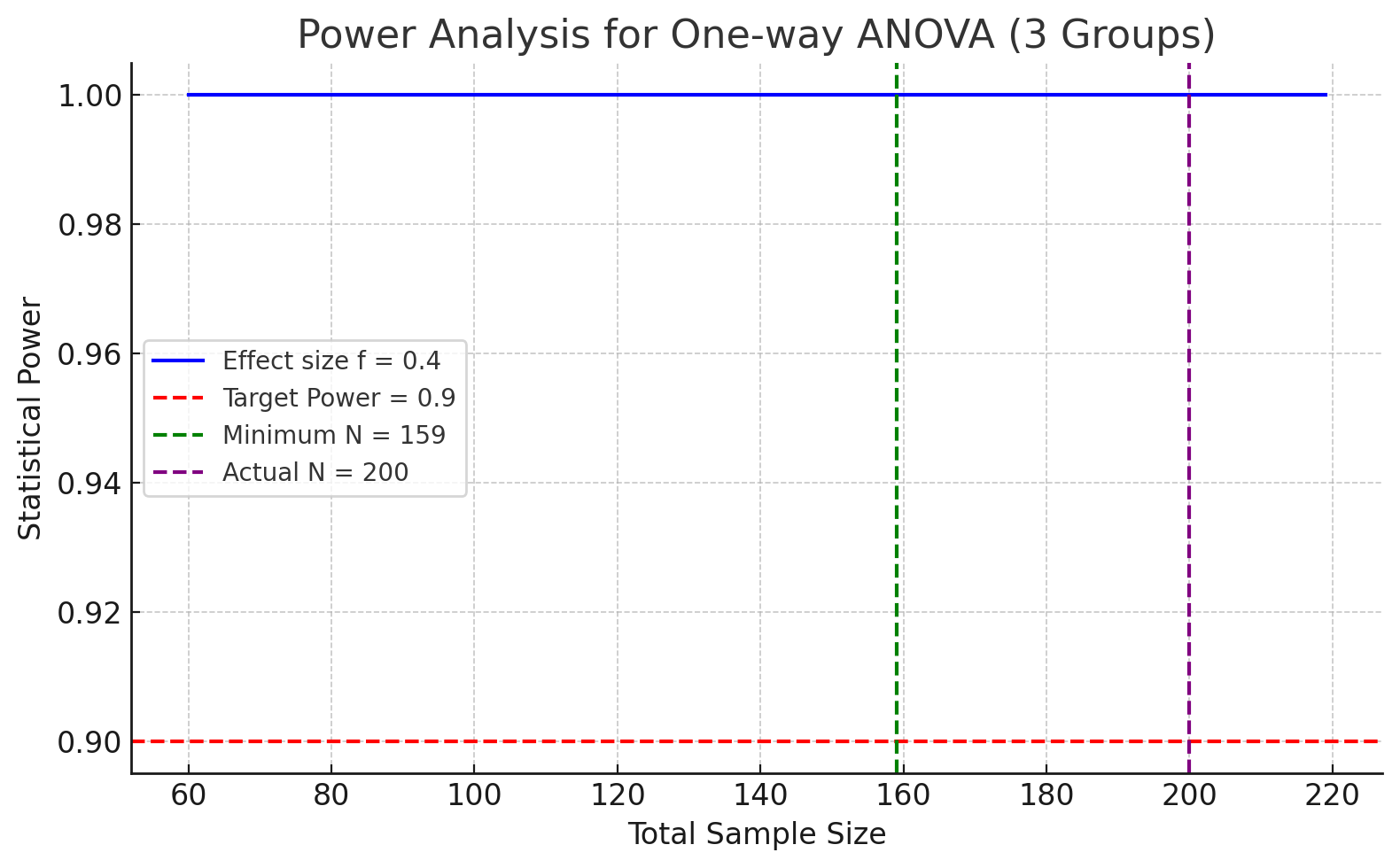


**Figure S1.** Sample size estimation using G*Power 3.1. Power curve for detecting differences among three groups using one-way ANOVA with effect size f = 0.4, α = 0.05, and desired power = 0.9. The vertical green line indicates the estimated minimum sample size (n = 159), and the red dashed line indicates the desired power threshold (0.9)
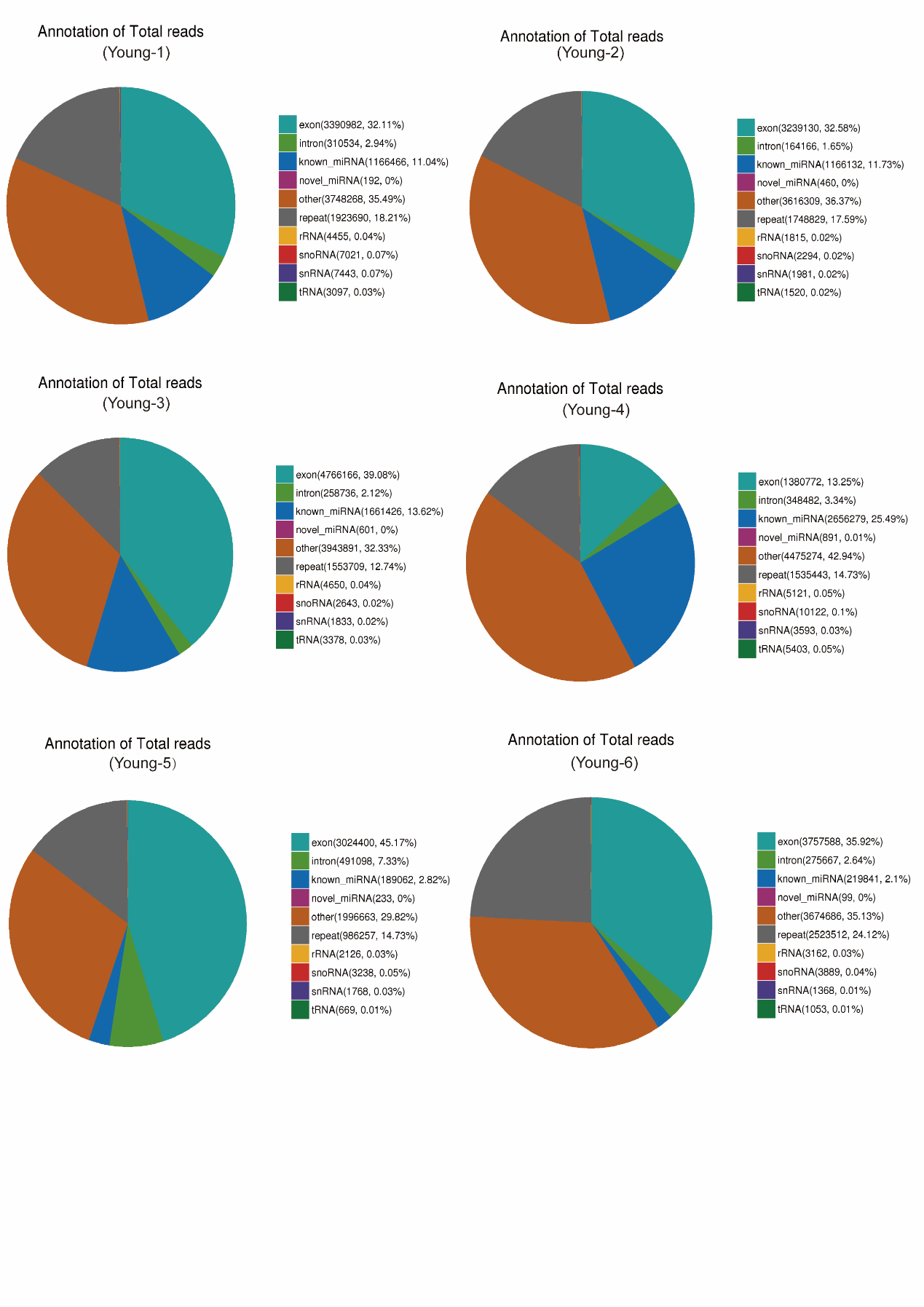


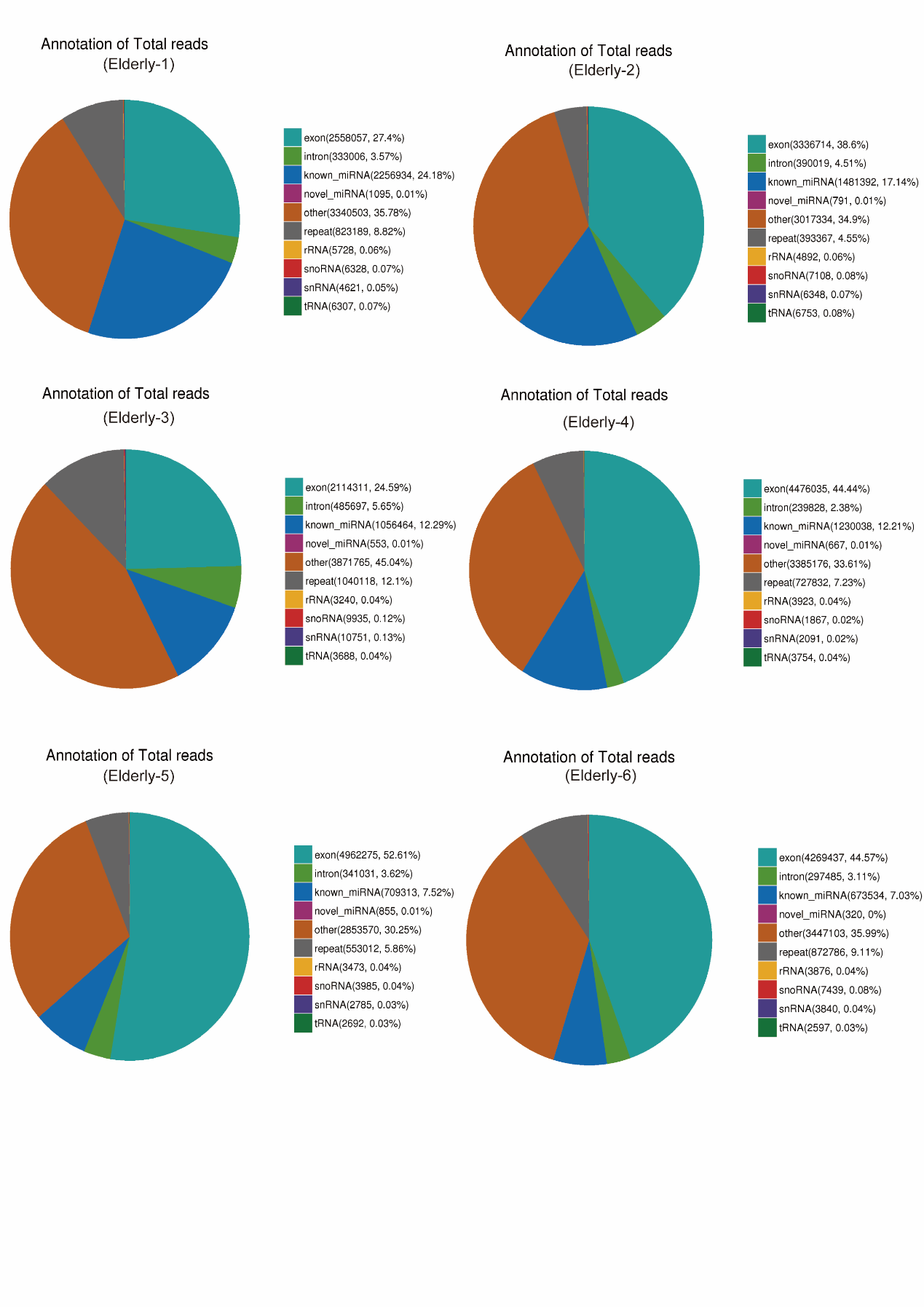


**Figure S2.** Proportions of various types of small RNAs in plasma exosomes from six elderly individuals and six adults.

**
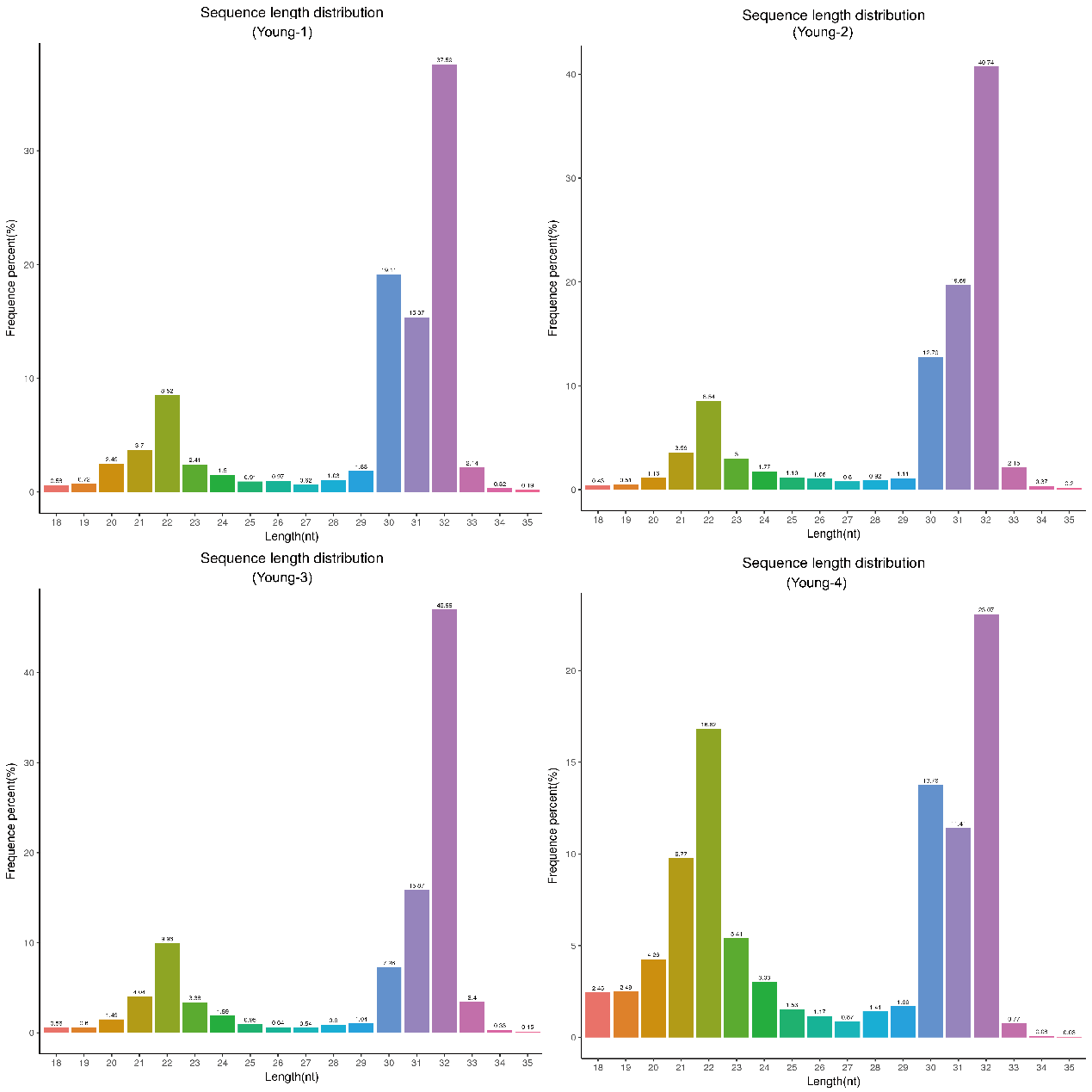

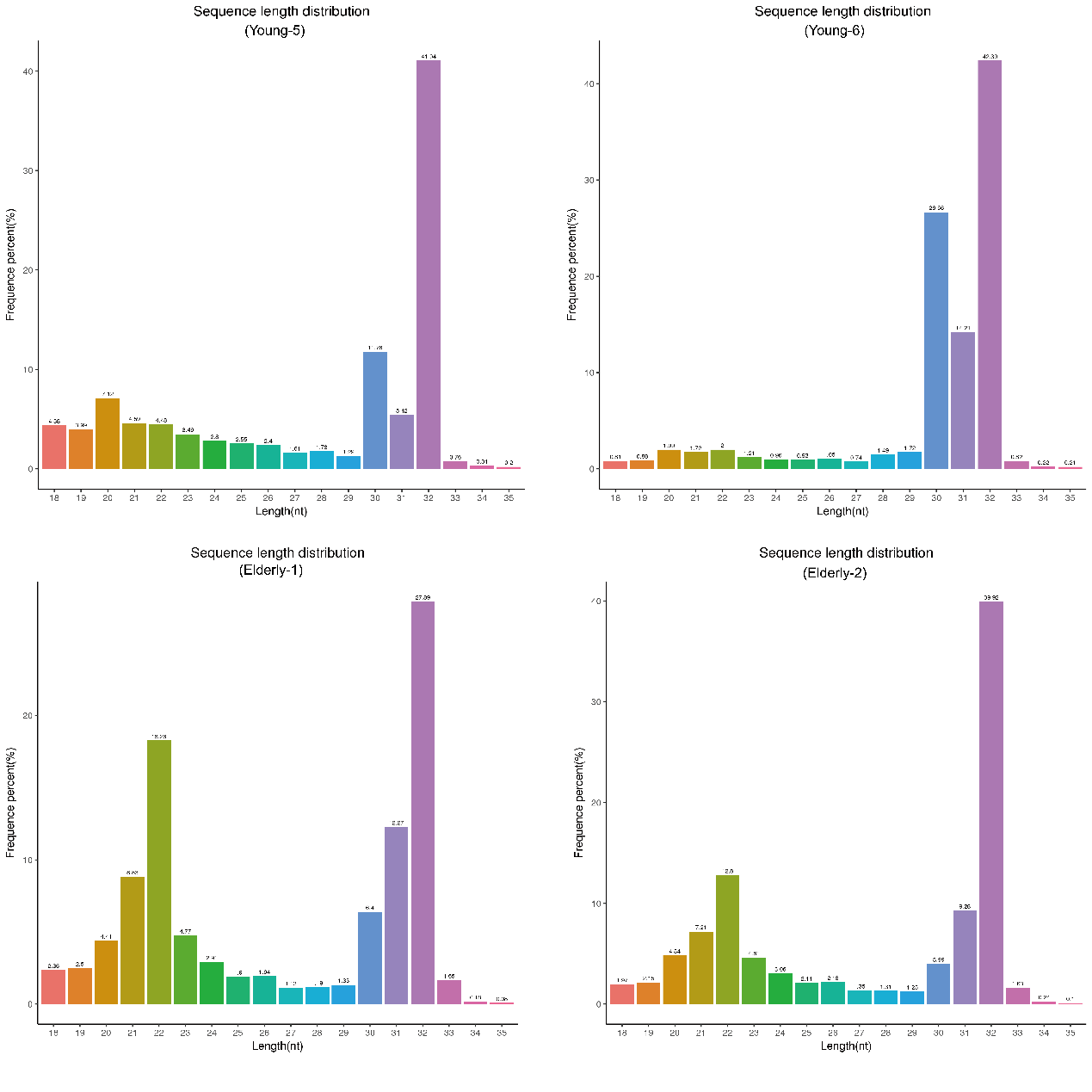

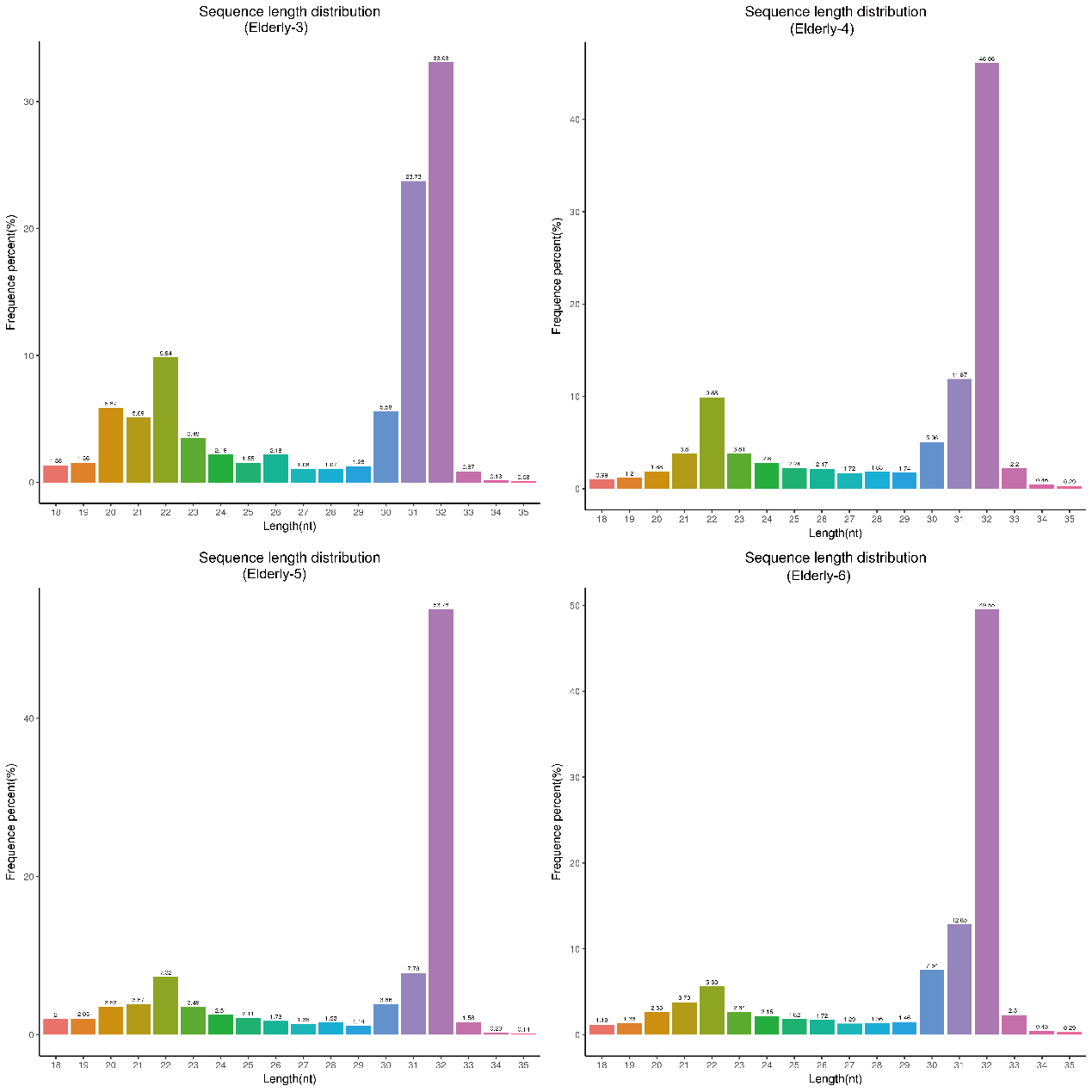
 Figure S3.** Length distribution of serum exosomal small RNAs from six elderly and six young individuals.


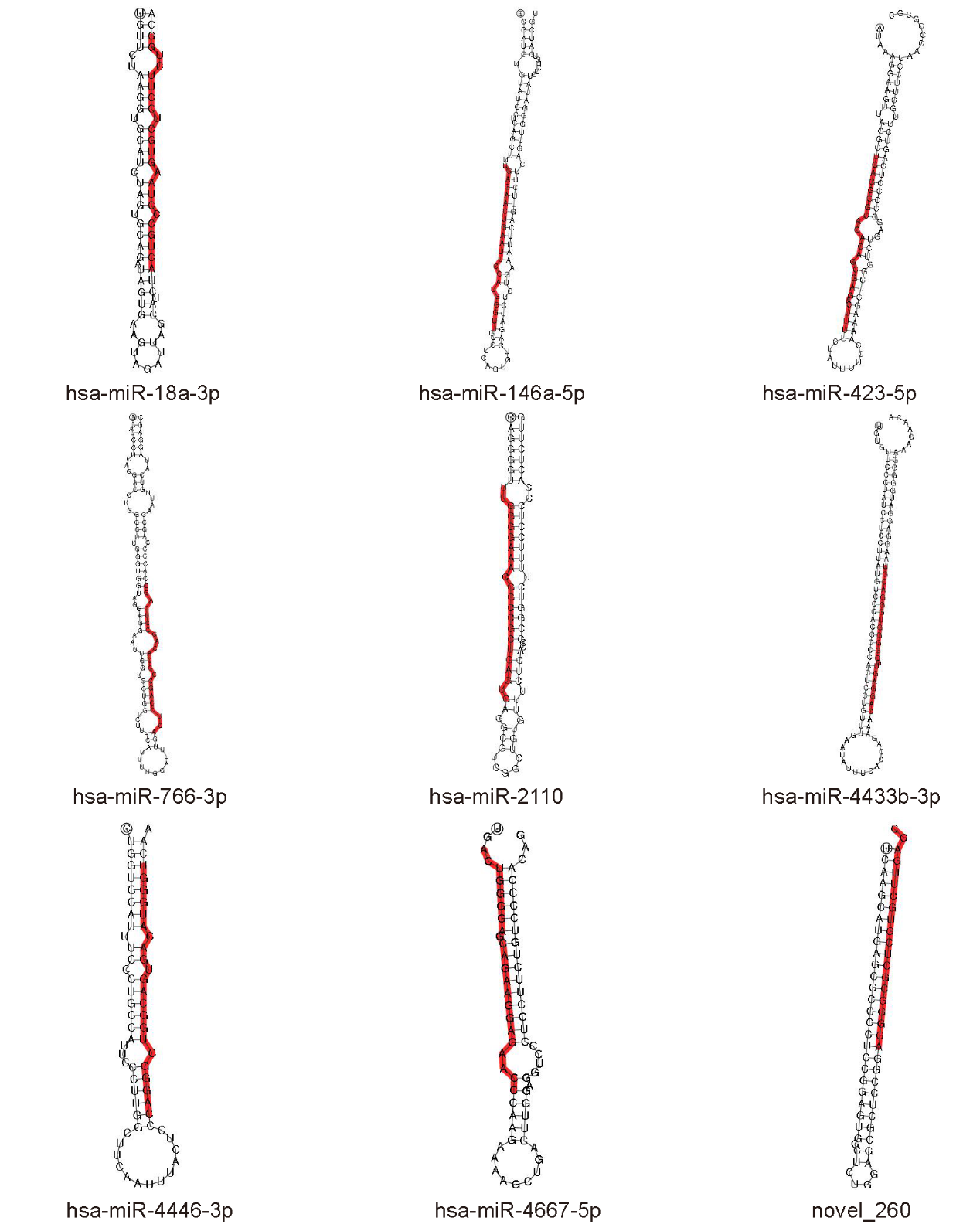


**Figure S4.** Predicted secondary structures of the nine differentially expressed miRNAs. The entire structure represents the precursor miRNA, and the red-highlighted region denotes the location of the mature miRNA sequence.


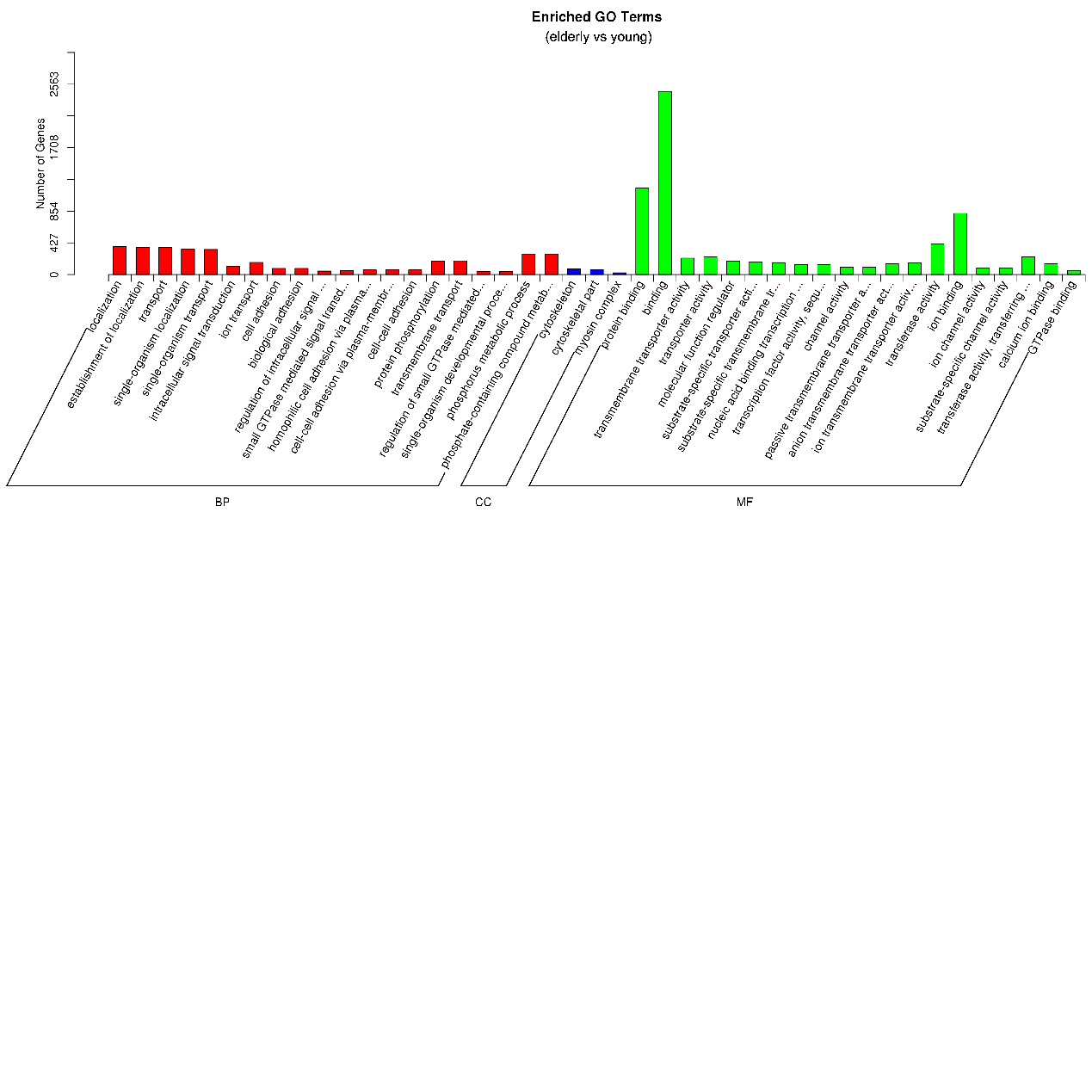


**Figure S5.** Gene ontology (GO) enrichment analysis based on differentially expressed genes (DEGs) between elderly vs. young individuals, showing GO terms in three categories, i.e., biological process (BP), cellular component (CC), and molecular function (MF).


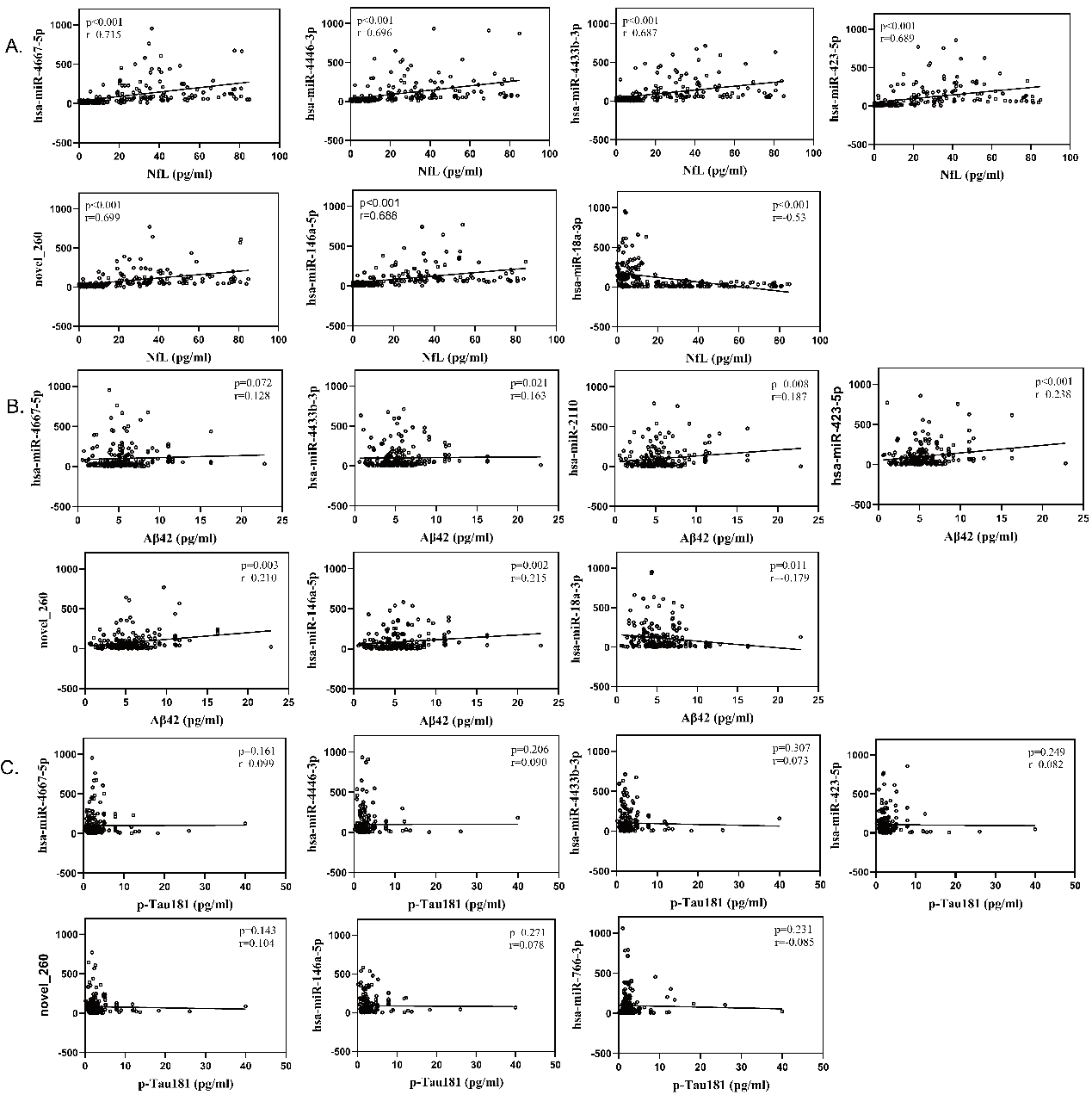

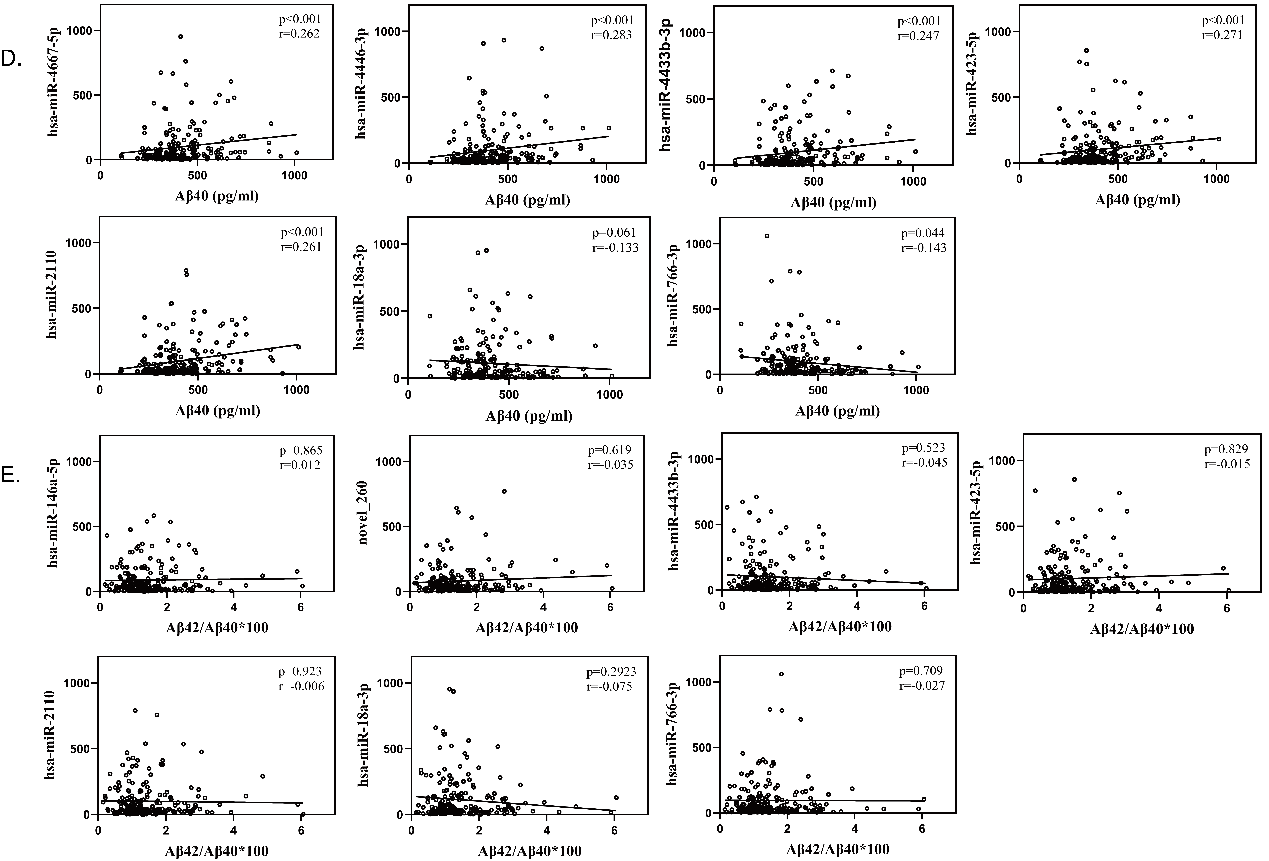


**Figure S6.** Spearman correlation analysis between plasma exosomal miRNA expression (measured in arbitrary units, AU) and plasma biomarkers related to brain aging, including **A.** neurofilament light chain (NfL; pg/ml), **B.** amyloid-beta 42 (Aβ42; pg/ml), **C.** phosphorylated Tau181 (p-Tau181; pg/ml), **D.** amyloid-beta 40 (Aβ40; pg/ml), and **E.** the ratio of Aβ42 to Aβ40. Spearman correlation coefficient (*r*) and *p-*values are indicated for each panel.

**Table S1.** Multivariable regression analysis assessing the association between plasma exosomal miRNA level and plasma brain aging biomarkers.

| Aβ42/Aβ40*100 | Model B | *p*–*value* | 0.40356 | 0.19223 | 0.6088 | 0.76246 | 0.39204 | 0.22127 | 0.35767 | 0.57616 | 0.94446 | 0.63431 |
| --- | --- | --- | --- | --- | --- | --- | --- | --- | --- | --- | --- | --- |
|  |  | *β (95%CI)* | -7.706 (-25.748, 10.335) | -12.918 (-32.262, 6.426) | 5.176 (-14.611, 24.962) | 3.383 (-18.523, 25.289) | -8.889 (-29.196, 11.418) | -11.490 (-29.837, 6.858) | -7.926 (-24.772, 8.920) | -4.223 (-19.005, 10.558) | 0.711 (-19.267, 20.689) | 3.779 (-11.767, 19.325) |
|  | Model A | *p*–*value* | 0.37941 | 0.26258 | 0.62172 | 0.919 | 0.31595 | 0.09254 | 0.32363 | 0.80283 | 0.7948 | 0.4761 |
|  |  | *β (95%CI)* | -7.868 (-25.374, 9.637) | -11.145 (-30.587, 8.297) | 4.721 (-14.003, 23.445) | 1.102 (-20.115, 22.319) | -10.230 (-30.175, 9.714) | -15.948 (-34.439, 2.544) | -8.130 (-24.235, 7.974) | -1.853 (-16.379, 12.673) | 2.580 (-16.839, 22.000) | 5.576 (-9.731, 20.883) |
|  | Non-adjusted | *p*–*value* | 0.79323 | 0.14647 | 0.95752 | 0.62031 | 0.66969 | 0.31286 | 0.6871 | 0.78013 | 0.49856 | 0.28756 |
|  |  | *β (95%CI)* | -2.749 (-23.280, 17.781) | -18.106 (-42.449, 6.237) | -0.593 (-22.373, 21.188) | 5.945 (-17.539, 29.430) | -4.965 (-27.743, 17.813) | -10.960 (-32.190, 10.270) | -3.802 (-22.275, 14.671) | 2.436 (-14.647, 19.520) | 7.652 (-14.470, 29.774) | 9.380 (-7.861, 26.622) |
| Aβ40 (pg/ml) | Model B | *p*–*value* | 0.09222 | 0.05624 | 0.97796 | 0.23954 | 0.61592 | 0.89952 | 0.73006 | 0.44351 | 0.88231 | 0.99616 |
|  |  | *β (95%CI)* | 0.100 (-0.016, 0.216) | 0.122 (-0.002, 0.246) | -0.002 (-0.130, 0.126) | 0.085 (-0.056, 0.226) | 0.034 (-0.098, 0.165) | 0.008 (-0.111, 0.127) | 0.019 (-0.090, 0.128) | 0.037 (-0.058, 0.133) | -0.010 (-0.139, 0.119) | -0.000 (-0.101, 0.100) |
|  | Model A | *p*–*value* | 0.15181 | 0.04975 | 0.90757 | 0.44126 | 0.7343 | 0.61682 | 0.97787 | 0.25086 | 0.94128 | 0.38208 |
|  |  | *β (95%CI)* | 0.079 (-0.029, 0.187) | 0.120 (0.001, 0.240) | 0.007 (-0.109, 0.123) | 0.052 (-0.080, 0.183) | 0.021 (-0.102, 0.145) | 0.029 (-0.086, 0.145) | -0.001 (-0.101, 0.099) | 0.053 (-0.037, 0.142) | 0.005 (-0.116, 0.125) | 0.042 (-0.052, 0.137) |
|  | Non-adjusted | *p*–*value* | 0.00074 | 0.30944 | 0.04167 | 0.01303 | 0.02147 | 0.01414 | 0.04389 | 0.00159 | 0.03786 | 0.00702 |
|  |  | *Β (95%CI)* | 0.207 (0.089, 0.326) | -0.075 (-0.220, 0.070) | -0.134 (-0.262, -0.006) | 0.176 (0.038, 0.313) | 0.158 (0.024, 0.291) | 0.157 (0.033, 0.282) | 0.112 (0.004, 0.221) 0.04389 | 0.162 (0.063, 0.260) | 0.139 (0.009, 0.269) | 0.140 (0.039, 0.241) |
| p-tau181(pg/ml) | Model B | *p*–*value* | 0.72803 | 0.14888 | 0.97974 | 0.97887 | 0.65871 | 0.32242 | 0.8588 | 0.98401 | 0.98042 | 0.97051 |
|  |  | *β (95%CI)* | 0.751 (-3.476, 4.978) | -3.343 (-7.864, 1.177) | -0.060 (-4.692, 4.572) | 0.069 (-5.057, 5.195) | -1.074 (-5.832, 3.684) | -2.176 (-6.474, 2.122) | 0.359 (-3.591, 4.309) | -0.035 (-3.496, 3.425) | 0.059 (-4.615, 4.732) | -0.069 (-3.708, 3.570) |
|  | Model A | *p*–*value* | 0.6053 | 0.09309 | 0.87584 | 0.79993 | 0.78676 | 0.42617 | 0.64031 | 0.59921 | 0.62856 | 0.44369 |
|  |  | *β (95%CI)* | 1.064 (-2.966, 5.095) | -3.834 (-8.287, 0.619) | -0.344 (-4.652, 3.964) | -0.632 (-5.510, 4.247) | -0.635 (-5.233, 3.962) | -1.740 (-6.016, 2.536) | 0.886 (-2.825, 4.596) | -0.897 (-4.235, 2.442) | -1.103 (-5.567, 3.360) | -1.378 (-4.897, 2.141) |
|  | Non-adjusted | *p*–*value* | 0.45878 | 0.09237 | 0.66802 | 0.98238 | 0.96707 | 0.67928 | 0.49077 | 0.88753 | 0.88513 | 0.68328 |
|  |  | *β (95%CI)* | 1.788 (-2.933, 6.510) | -4.828 (-10.423, 0.767) | -1.099 (-6.111, 3.914) | 0.061 (-5.350, 5.472) | 0.111 (-5.137, 5.358) | -1.035 (-5.934, 3.864) | 1.497 (-2.753, 5.747) | -0.284 (-4.219, 3.650) | -0.376 (-5.476, 4.723) | -0.830 (-4.809, 3.150) |
| Aβ42 (pg/ml) | Model B | *p*–*value* | 0.80382 | 0.807 | 0.43922 | 0.12107 | 0.18159 | 0.07317 | 0.32501 | 0.49149 | 0.61994 | 0.5707 |
|  |  | *β (95%CI)* | 0.750 (-5.160, 6.660) | 0.793 (-5.561, 7.148) | 2.557 (-3.907, 9.021) | 5.656 (-1.461, 12.774) | -4.530 (-11.151, 2.091) | -5.491 (-11.462, 0.481) | -2.773 (-8.280, 2.734) | -1.699 (-6.531, 3.132) | 1.655 (-4.874, 8.183) | 1.473 (-3.609, 6.555) |
|  | Model A | *p*–*value* | 0.90457 | 0.6314 | 0.39979 | 0.27756 | 0.10008 | 0.02352 | 0.25813 | 0.98407 | 0.42464 | 0.16496 |
|  |  | *β (95%CI)* | 0.339 (-5.199, 5.877) | 1.509 (-4.646, 7.663 | 2.542 (-3.363, 8.447) | 3.710 (-2.969, 10.389) | -5.285 (-11.555, 0.984) | -6.760 (-12.564, -0.955) | -2.940 (-8.021, 2.141) | ’-0.047 (-4.634, 4.540) | 2.499 (-3.623, 8.622) | 3.424 (-1.391, 8.240) |
|  | Non-adjusted | *p*–*value* | 0.01829 | 0.02455 | 0.12839 | 0.00465 | 0.47694 | 0.82272 | 0.24151 | 0.02314 | 0.00555 | 0.00129 |
|  |  | *β (95%CI)* | 7.411 (1.306, 13.515) | -8.421 (-15.705, -1.136) | -5.086 (-11.614, 1.443) | 10.140 (3.197, 17.082) | 2.495 (-4.367, 9.356) | 0.734 (-5.682, 7.151) | 3.328 (-2.225, 8.880) | 5.938 (0.853, 11.023) | 9.369 (2.820, 15.917) | 8.456 (3.378, 13.534) |
| Nfl (pg/ml) | Model B | *p*–*value* | 0.90984 | 0.00768 | 0.01688 | 0.04456 | 0.67567 | 0.58353 | 0.74651 | 0.5006 | 0.08951 | 0.69416 |
|  |  | *β (95%CI)* | 0.093 (-1.515, 1.701) | 2.332 (0.637, 4.027) | 2.133 (0.400, 3.867) | 1.989 (0.062, 3.917) | 0.387 (-1.422, 2.196) | -0.459 (-2.096, 1.179) | -0.248 (-1.750, 1.254) | -0.453 (-1.767, 0.862) | -1.536 (-3.299, 0.227) | 0.278 (-1.105, 1.661) |
|  | Model A | *p*–*value* | 0.5845 | 0.00009 | 0.05821 | 0.22297 | 0.61887 | 0.79852 | 0.60545 | 0.97206 | 0.26494 | 0.25684 |
|  |  | *β (95%CI)* | 0.379 (-0.978, 1.737) | 2.963 (1.510, 4.416) | 1.398 (-0.040, 2.836) | 1.021 (-0.616, 2.659) | 0.394 (-1.155, 1.942) | -0.188 (-1.631, 1.255) | -0.330 (-1.580, 0.920) | 0.020 (-1.105, 1.146) | -0.856 (-2.356, 0.644) | 0.687 (-0.497, 1.870) |
|  | Non-adjusted | *p*–*value* | <0.00001 | <0.00001 | <0.00001 | <0.00001 | <0.00001 | <0.00001 | <0.00001 | <0.00001 | <0.00001 | <0.00001 |
|  |  | *β (95%CI)* | 2.740 (2.023, 3.457) | -2.872 (-3.754, -1.991) | -2.387 (-3.182, -1.592) | 2.783 (1.939, 3.628) | 2.814 (2.003, 3.626) | 2.507 (1.741, 3.273) | 2.145 (1.478, 2.812) | 2.229 (1.629, 2.830) | 2.419 (1.610, 3.227) | 2.167 (1.553, 2.780) |
|  |  |  | hsa-miR-2110 | has-miR-18a-3p | hsa-miR-766-3p | hsa-miR-4446-3p | hsa-miR-4667-5p | hsa-miR-4433b-3p | hsa-146a-5p | hsa-146a-5p-1 | hsa-miR-423-5p | novel_260 |
